# Inhibition of Specific NF-κB Activity Contributes to the Tumor Suppressor Function of 14-3-3σ in Breast Cancer

**DOI:** 10.1371/journal.pone.0038347

**Published:** 2012-05-31

**Authors:** Julia Inglés-Esteve, Mònica Morales, Alba Dalmases, Ricard Garcia-Carbonell, Alba Jené-Sanz, Núria López-Bigas, Mar Iglesias, Cristina Ruiz-Herguido, Ana Rovira, Federico Rojo, Joan Albanell, Roger R. Gomis, Anna Bigas, Lluís Espinosa

**Affiliations:** 1 Cancer Research Program, IMIM (Hospital del Mar Research Institute), Barcelona, Spain; 2 Institut de Recerca Biomèdica (IRB), Barcelona, Spain; 3 Medical Oncology Service, Hospital del Mar, Barcelona, Spain; 4 Department of Pathology, Hospital del Mar, Barcelona, Spain; 5 Research Unit on Biomedical Informatics, Department of Experimental and Health Sciences, Universitat Pompeu Fabra, Barcelona, Spain; 6 Institució Catalana de Recerca i Estudis Avançats (ICREA), Barcelona, Spain; 7 Pathology Department, IIS-Fundacion Jimenez Diaz, Madrid, Spain; 8 Universitat Pompeu Fabra, Barcelona, Spain; The Chinese University of Hong Kong, Hong Kong

## Abstract

14-3-3σ is frequently lost in human breast cancers by genetic deletion or promoter methylation. We have now investigated the involvement of 14-3-3σ in the termination of NF-κB signal in mammary cells and its putative role in cancer relapse and metastasis. Our results show that 14-3-3σ regulates nuclear export of p65-NF-κB following chronic TNFα stimulation. Restoration of 14-3-3σ in breast cancer cells reduces migration capacity and metastatic abilities *in vivo*. By microarray analysis, we have identified a genetic signature that responds to TNFα in a 14-3-3σ-dependent manner and significantly associates with different breast and other types of cancer. By interrogating public databases, we have found that over-expression of this signature correlates with poor relapse-free survival in breast cancer patients. Finally, screening of 96 human breast tumors showed that NF-κB activation strictly correlates with the absence of 14-3-3σ and it is significantly associated with worse prognosis in the multivariate analysis. Our findings identify a genetic signature that is important for breast cancer prognosis and for future personalized treatments based on NF-κB targeting.

## Introduction

Breast cancer is the most common cancer that affects women in western countries, and metastasis is a fatal consequence of this disease [1]. The identification of the mechanisms involved in the survival of breast cancer cells and their ability to colonize distant tissues is a crucial issue for personalized cancer therapy and improvement of patient treatment. However, complexities of the cellular interactions and the molecular components that are involved in breast cancer progression and metastasis have challenged the significance of new discoveries in this field [2]. A majority of the metastases occurring in breast cancer patients arise in the bone and are generally osteolytic, involving the mobilization of osteoclasts that cause bone resorption, with severe pain, bone fractures, nerve compression and hypercalcemia [3], [4], [5]. Interestingly, a particular characteristic of the bone microenvironment is not only that it represents a bona-fide stem cell niche for hematopoietic cells, but also that it contains immune cells that are a source of inflammatory cytokines. Inflammation is a major contributor to carcinogenesis, being Nuclear Factor-κB (NF-κB) the master transducer of inflammatory signals [6]. Breast cancer has repetitively been linked to high NF-κB activity [7], [8], [9], [10], [11], [12], which regulates genes associated with tumor invasion and metastasis such as Maspin [13] and GM-CSF [9]. NF-κB activation is triggered by different extracellular stimuli such as inflammatory cytokines, EGF or RANKL [14], [15], [16], [17], a finding that is the base for using antibodies against TNFα, soluble TNFα receptors or RANKL inhibitors in patients with bone-metastasis [18], [19]. However, alterations in inhibitory factors of NF-κB signal also contribute in maintaining high NF-κB activity in cancer cells [20].

14-3-3 is a highly conserved family of adaptor proteins that regulate multiple signal transduction pathways by regulating activity (e.g. Raf, JNK) [21], [22] or promoting the nuclear export of pre-phosphorylated substrates (e.g. FOXO, CDC25, HDAC or regulators of mTOR) [23], [24], [25], [26]. We previously demonstrated that different 14-3-3 members bind and facilitate nuclear export of NF-κB complexes after TNFα stimulation [27]. Rapid nuclear export of NF-κB (mainly the p65/p50 dimers) by newly synthesized IκBα is essential for the post-induction repression phase of the NF-κB signaling [28], [29]. Interestingly, 14-3-3σ has been categorized as a tumor suppressor in human breast cancers where is frequently lost due to gene deletion or promoter methylation [30], [31], [32], [33]. We have now investigated the role of 14-3-3σ in breast cancer cells and its putative involvement in the metastatic phenotype. We have found that 14-3-3σ levels determine the kinetics of nuclear p65 export after TNFα stimulation and identified a genetic signature that responds to TNFα in a 14-3-3σ-dependent manner. This signature is significantly associated with breast, intestinal, ovarian and lung cancer in humans, and its over-expression correlates with poor relapse-free survival breast cancer patients. Importantly, manipulation of 14-3-3σ levels in mammary cells leads to changes in cell migration *in vitro* and affects the metastatic capacity of MDA-MB-231 breast cancer cells in mice. Finally, NF-κB activation is primarily found in breast tumors lacking 14-3-3σ expression and is significantly associated with reduced disease-free survival of the patients in uni- and multivariate analysis.

## Results

### Breast cancer cells with low levels of 14-3-3σ show delayed p65 nuclear export and increased NF-κB activity

**Figure 1:**
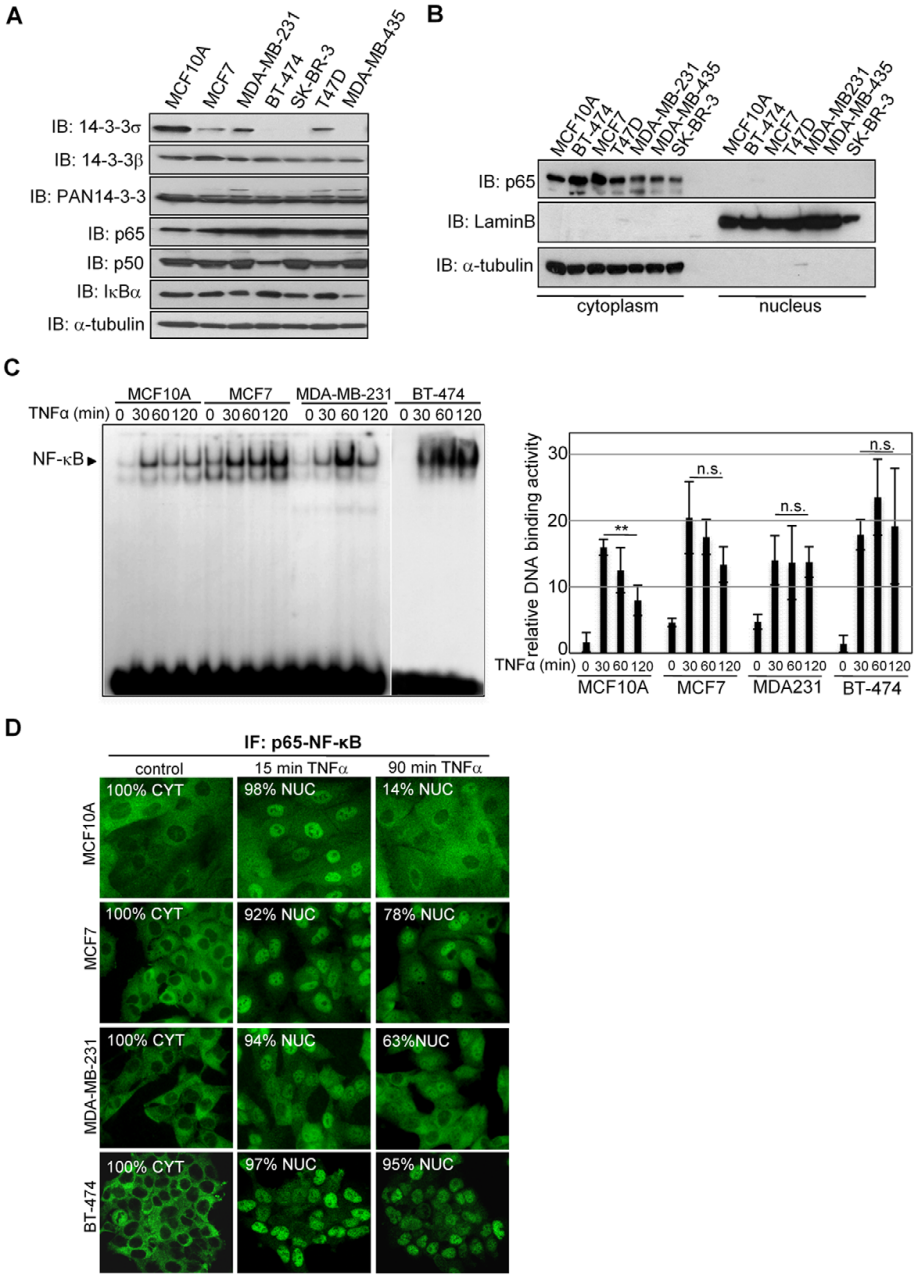
Breast cancer cells with low levels of 14-3-3σ show a delay in p65 nuclear export following chronic NF-κB activation. (A) Western blot analysis of 14-3-3σ expression in normal and cancer cell lines. (B) Subcellular fractionation from different mammary cell lines and western blot analysis with the indicated antibodies. (C) (left panel) TNFα-dependent activation of NF-κB in the indicated cell lines measured by EMSA and (right panel) densitometric analysis of four independent experiments (average and standard deviation). DNA-binding activity is represented relative to untreated MCF10A. (D) IF with specific α-p65 antibody of the indicated cells incubated with TNFα at different time points. NUC indicates cells containing nuclear p65 and CYT, exclusive cytoplasmic staining. A representative of three independent experiments is shown in all cases and all samples were equally processed.

To study whether 14-3-3σ was involved in NF-κB regulation in breast cancer cells, we first determined its expression levels in non-transformed MCF10A and breast cancer (MCF7, MDA-MB-231, BT-474, SK-BR-3 and T47D) cells. We found that 14-3-3σ is downregulated in cancer cells compared to MCF10A, whereas other 14-3-3 isoforms show comparable levels. 14-3-3σ was also absent from MDA-MB-435, previously considered as a breast cancer cells. In contrast, p65 and p50 NF-κB members and their negative regulator IκBα were similarly expressed in all tested cell lines (Figure 1A). However, we did not detect any nuclear p65 in non-stimulated breast cancer cells (Figure 1B).

Since we previously found that 14-3-3 participates in the post-activation repression of NF-κB [27], we now tested whether reduced 14-3-3σ levels in breast cancer cells affects NF-κB activation or signal duration. By Electrophoretic Mobility Shift Assay (EMSA) using specific κB probe, we found sustained nuclear NF-κB activity in MCF7 and BT-474 and to a minor extent in MDA-MB-231 breast cancer cells compared to MCF10A cells after TNFα treatment (Figure 1C). Next, we determined whether these changes were associated with the capacity of these cells to retain p65 in the nucleus. By immunofluorescence (IF), we found that MCF7, MDA-MB-231 and BT-474 cells showed a delay in redistributing nuclear p65 to the cytoplasm compared with MCF10A (78%, 63% and 95% of cells containing nuclear p65 compared with 14% in MCF10A cells after 90 min with TNFα) (Figure 1D). Specificity control for p65 staining was performed using p65-deficient cells (Figure S1).

### p65 binds to 14-3-3σ in mammary cells in a TNFα-dependent manner

**Figure 2:**
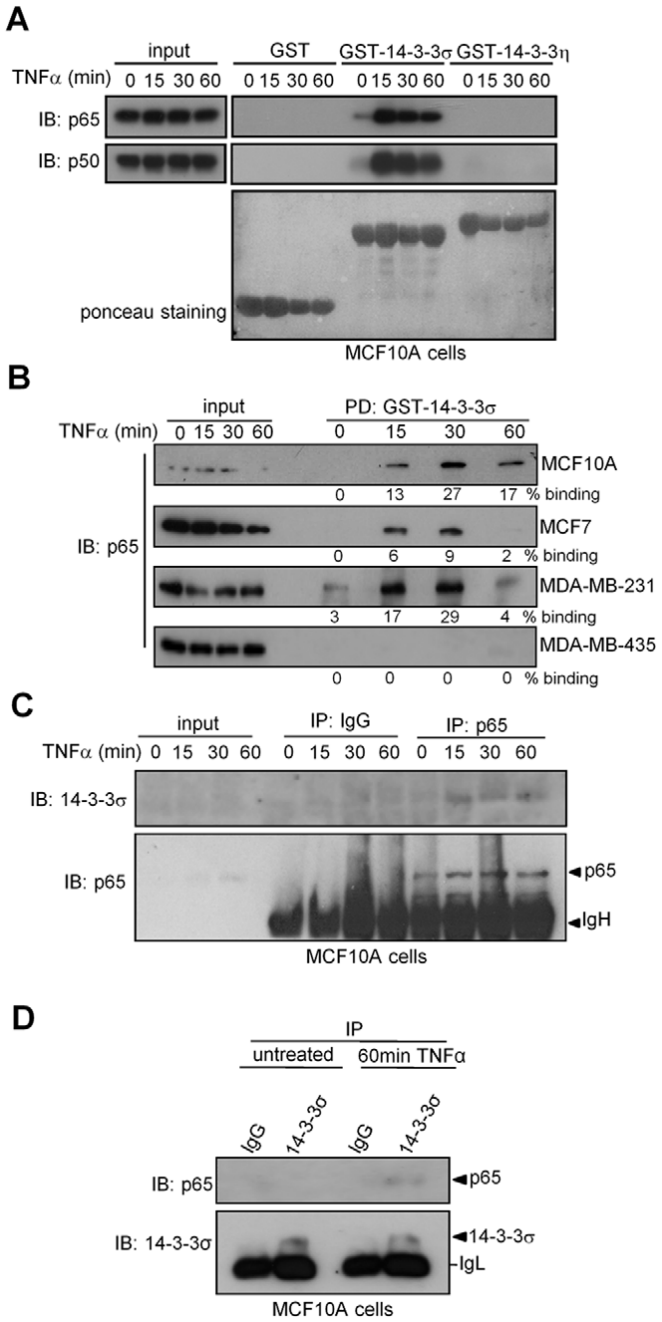
p65 preferentially binds to 14-3-3σ in normal and breast cancer cells following NF-κB activation. (A, B) Pull down experiment using different GST fusion proteins and cell extracts from (A) TNFα-treated MCF10 and (B) different breast cancer cell lines. Densitometric quantification of binding is shown below each panel. (C–D) Co-immunoprecipitation of endogenous proteins from MCF10A (C) immunoprecipitation of p65 and detection of 14-3-3σ by western blot and (D) immunoprecipitation of 14-3-3σ and detection of p65.

We previously showed that TNFα induces p65 binding to 14-3-3β and 14-3-3η in HEK-293T cells [27]. However, the fact that 14-3-3σ deficiency in breast cancer cells correlates with delayed p65 nuclear export suggests a non-redundant function for this isoform in mammary cells. By pull-down (PD) we confirmed that both p65 and p50 isolated from MCF10A cells bound GST-14-3-3σ in response to TNFα. Moreover, this interaction was isoform-specific since both NF-κB proteins failed to bind 14-3-3η in the same experiment (Figure 2A). Comparable results were obtained using cell extracts from different breast cancer cells but not from MDA-MB-435 (Figure 2B). By coprecipitation experiments we demonstrated that endogenous 14-3-3σ associates with p65 in response to TNFα in non-transformed mammary cells (Figure 2C and 2D). Although we cannot formally conclude that the interaction between 14-3-3σ and p65 is direct, the presence of three 14-3-3-binding sites in the p65 protein [27] strongly suggests this possibility.

### Defective expression of 14-3-3σ is responsible for delayed p65 nuclear export in breast cancer cells

**Figure 3:**
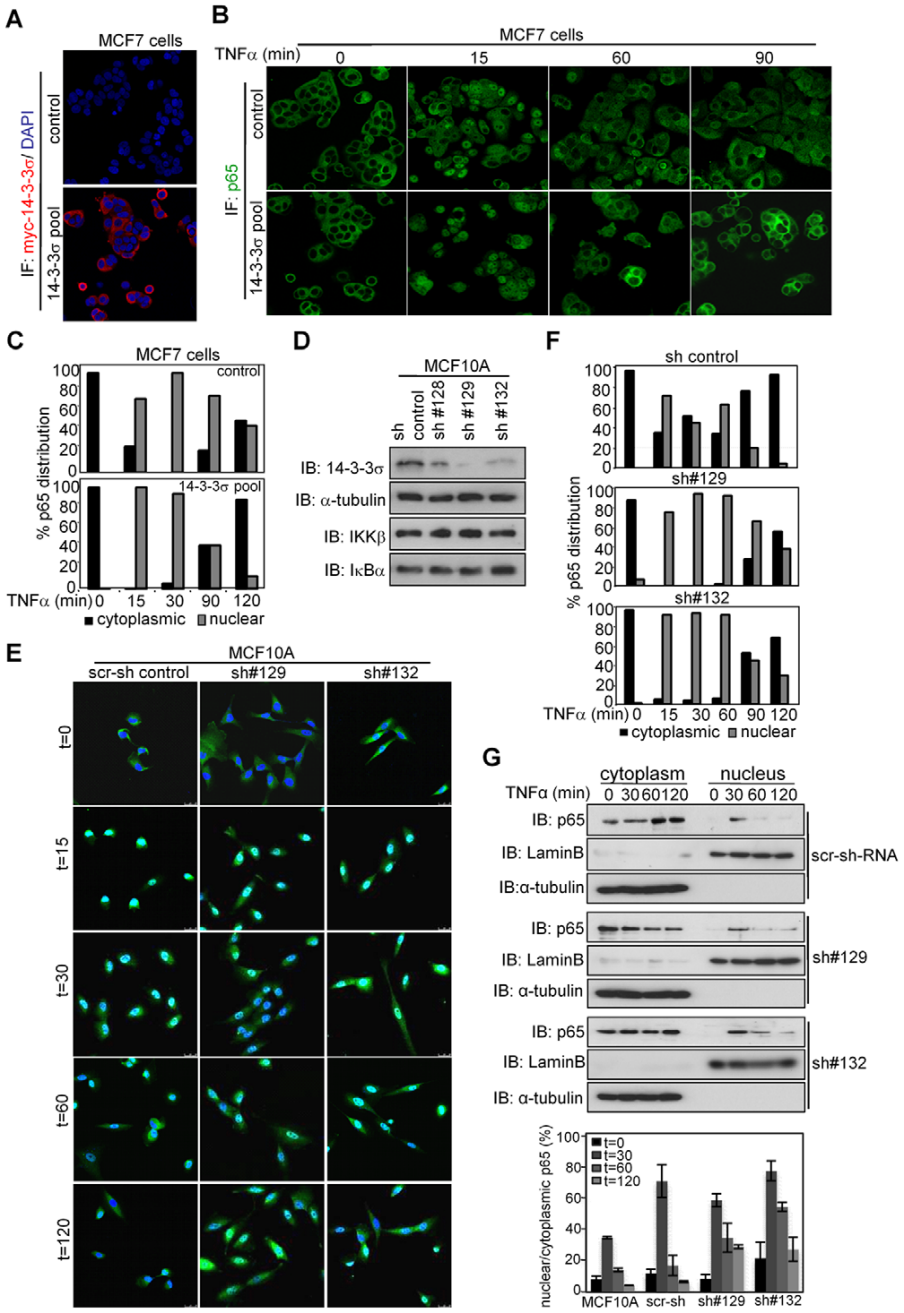
Defective 14-3-3σ is responsible for delayed p65 nuclear export in breast cancer cells. (A and B) IF analysis of 14-3-3σ expression (A) and p65-NF-κB nuclear translocation (B) in MCF7 control and 14-3-3σ expressing cells. (C) Quantification of p65 subcellular distribution in one representative of 3 independent experiments. (D) Western blot analysis of 14-3-3σ levels in MCF10A pools transduced with different shRNA. (E, F) Representative images of p65 IF (E) and quantification of p65 subcellular distribution in MCF10A cells (F). One representative of three independent experiments is shown. (G) Subcellular fractionation followed by western blot analysis of p65 in the different MCF10A pools transduced with scrambled or 14-3-3σ-shRNAs. Average of densitometric quantification of 3 independent experiments is shown in the lower panel. For representation of p65 distribution in 3c and 3f, two groups have been considered, cytoplasmic: homogenous or cytoplasmic staining and nuclear: preferentially nuclear distribution.

To determine whether 14-3-3σ deficiency was responsible for delayed nuclear export of p65 in breast cancer cells, we generated pools of MCF7 cells stably expressing the myc-14-3-3σ construct or the control vector (Figure 3A). By IF, we found that ectopic 14-3-3σ enhanced the ability of MCF7 cells to re-export p65 from the nucleus after TNFα stimulation (Figures 3B and 3C). Moreover, this effect was not due to changes in endogenous IκBα basal levels, TNFα-induced degradation of IκBα (that is essential for NF-κB activation) or IκBα re-synthesis (required for the post-activation repression of NF-κB) (Figure S2A). Identical results were obtained using MDA-MB-231 cells (Figures S3A, S3B and S3C) or MCF7 carrying a doxycycline-inducible 14-3-3σ construct (Figures S4A and S4B).

Next, to further study whether reduced levels of 14-3-3σ in tumor cells were responsible for delayed p65 cytoplasmic redistribution, we knocked-down 14-3-3σ in non-transformed MCF10A cells using three shRNA that resulted in different degrees of down-regulation (Figure 3D). Reduction of 14-3-3σ levels in MCF10A cells delayed or even blocked p65 export following chronic TNFα treatment (Figure 3E and 3F) without affecting IκBα levels (Figure 3D and S2B). These results were confirmed by subcellular fractionation of the different transduced cells (Figure 3G).

### An NF-κB-dependent transcriptional program depends on 14-3-3σ in breast cancer cells

**Figure 4:**
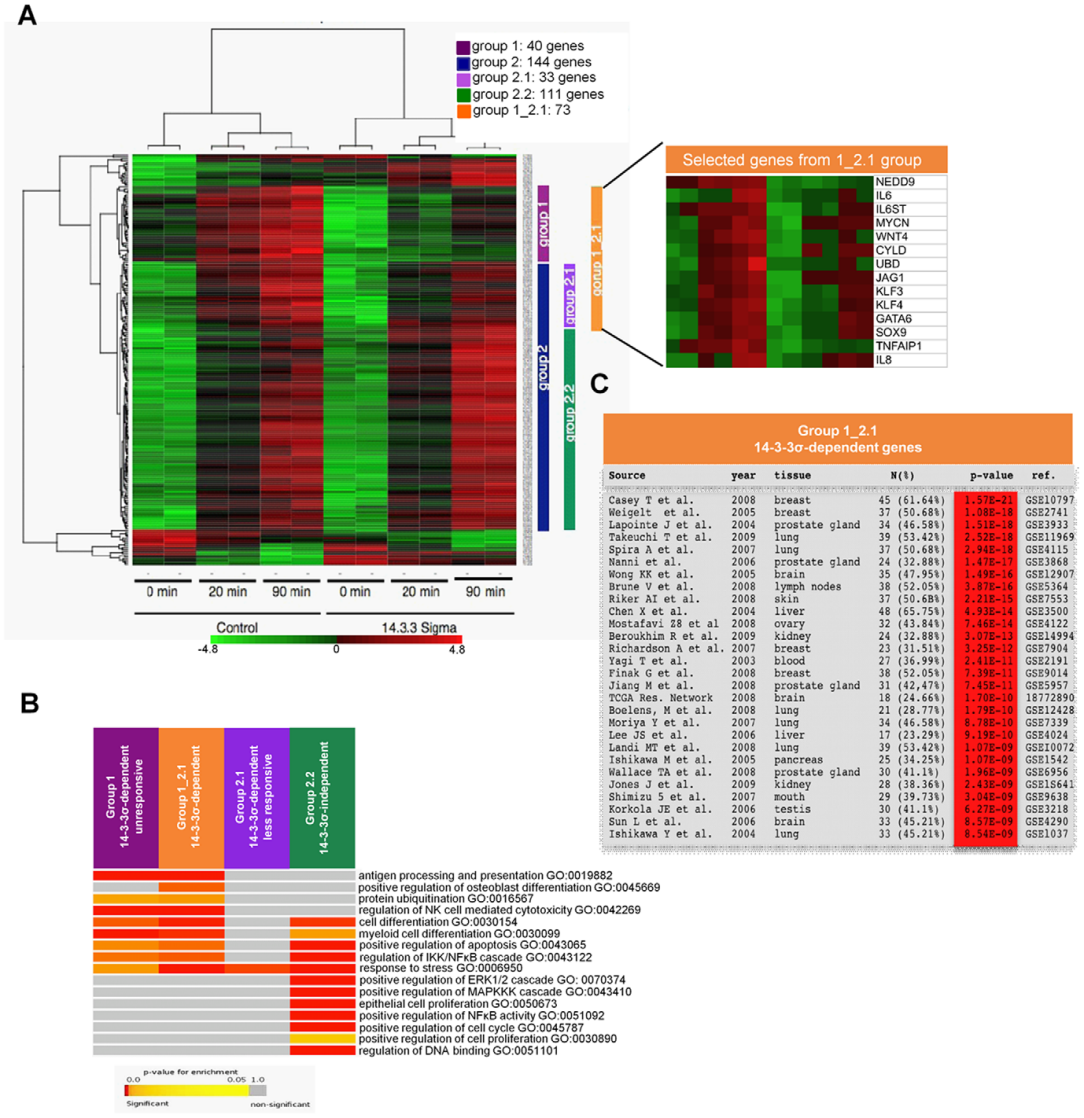
Ectopic expression of 14-3-3σ attenuates specific NF-κB-dependent activity in breast cancer cells. (A) Microarray expression analysis of MCF7 control or 14-3-3σ-expressing cells treated with TNFα at the indicated times. Group 1: genes induced by TNFα in control cells but not in 14-3-3σ-expressing MCF7 cells. Group 2: contains genes induced in both control and 14-3-3σ-expressing MCF7 cells, including differentially induced genes (group 2.1). A list including some of the genes identified in the 1_2.1 group (group 1 and 2.1) is shown in the right panel. (B) Selected Gene Ontology Biological Process terms in an enrichment analysis of 14-3-3σ-dependent and independent signatures. Colors towards red indicate significance; grey indicates no significant enrichment. (C) Enrichment analysis details of genes up-regulated in cancer samples of various experiments obtained using IntOGen software.

To investigate whether 14-3-3σ levels affect specific NF-κB dependent transcription in mammary cells, we performed expression microarray analysis of control or myc-14-3-3σ-expressing MCF7 cells treated with TNFα at different time points (GSE37139). We distinguished between 4 different groups of genes: genes induced by TNFα in control cells but not in 14-3-3σ-expressing MCF7 cells (group1); genes induced in both control and 14-3-3σ-expressing MCF7 cells (group 2), including differentially induced genes (group 2.1) or similarly induced genes (2.2). A fifth group called group 1_2.1 (group 1 plus group 2.1) contains all genes that were significantly upregulated in TNFα-treated control cells compared with 14-3-3σ-expressing MCF7 cells. Our results showed that TNFα also induced a similar transcriptional program in both types of cells (group 2.2), indicating the integrity of the pathway. Interestingly, a specific set of genes remained unresponsive (group 1), or less responsive (group 2.1) in 14-3-3σ-expressing cells (merged as a single 1_2.1 group for subsequent analysis) (Figure 4A). These 14-3-3σ-dependent genes include well-characterized NF-κB targets such as TNFAIP1, IL8, IL6 or GM-CSF (Figure 4A and S5). The differential response of some randomly selected 1_2.1 genes was confirmed by qRT-PCR (Figure S6). A comparable 14-3-3σ-dependent signature was identified in MDA-MB-231 cells (Figure S7). Furthermore, several 1_2.1 genes contained multiple NF-κB sites that clustered close to the transcription start site (TSS) (Figure S8), suggesting that they are direct NF-κB targets. In this sense, we found that 36 out of 73 genes contained in the 1_2.1 group were significantly downregulated in p65-knockdown breast cancer cell experiments available from public databases (GSE 31912 and GSE30670) (data not shown).

To ask whether the genes in different groups are functionally related, we performed enrichment analysis with Gene Ontology terms in the four groups. We found that genes belonging to groups 1 and 1_2.1 are enriched for genes involved in antigen processing and presentation, protein ubiquitination and regulation of IKK, while the 14-3-3-independent group 2.2 is enriched in genes involved in cell cycle and MAPKKK cascade among others. Moreover, all TNF-responder groups contained genes involved in NF-κB signaling.

We were also interested in assessing whether genes in 14-3-3σ-dependent signature (group 1_2.1) are miss-regulated in cancer cells compared to normal cells. For that we used sets of genes significantly upregulated in different cancer genomic experiments as obtained from IntOGen [34]. Briefly, IntOGen contains a collection of independent cancer genomic datasets from different tumor types, which are processed to identify genes altered in a significant number of samples. We found that genes in this group are significantly miss-regulated in different types of tumors including breast, lung, prostate gland, brain, skin, liver and others (Figure 4C).

### 14-3-3σ expression modulates breast cancer cell motility through NF-κB

GO enrichment analysis indicated that 1_2.1 genes were included in a restricted number of functional categories including apoptosis, antigen presentation, and stress responses among others. Thus, we analyzed whether 14-3-3σ expression affects specific biological processes in breast cancer cells. By flow cytometry analysis, we found comparable proliferation or apoptotic ratios in control or 14-3-3σ-expressing cells (MCF7 or MDA-MB-231), untreated or treated with TNFα (Figure S9 and S10). In contrast, wound-healing experiments demonstrated that MDA-MB-231 cells expressing 14-3-3σ showed a reduced motility compared to the controls (Figure S11) that was comparable to cells treated with the NF-κB inhibitor BAY11-7082 (Figure S11 and S12). Conversely, TNFα promotes cell migration concomitant with nuclear p65 accumulation in the migratory cells (Figure S12). Similarly, NF-κB inhibition blocked the invasive capacity of MDA-MB-231 cells in trans-well assays (Figure S13). Next, we performed wound-healing experiments using control MCF10A cells and two pools of these cells stably expressing different shRNA against 14-3-3σ (#129 and #132). In these experiments, control shRNA-transduced MCF10A cells displayed very low migratory capacity. However, reduction of 14-3-3σ levels by shRNA was sufficient to induce cell migration (Figure S14).

These results suggest that 14-3-3σ regulates a specific transcriptional program, which in turn affects particular cellular capacities such as cell migration.

### Ectopic expression of 14-3-3σ reduces the metastatic capacities of MDA-MB-231 cells

**Figure 5:**
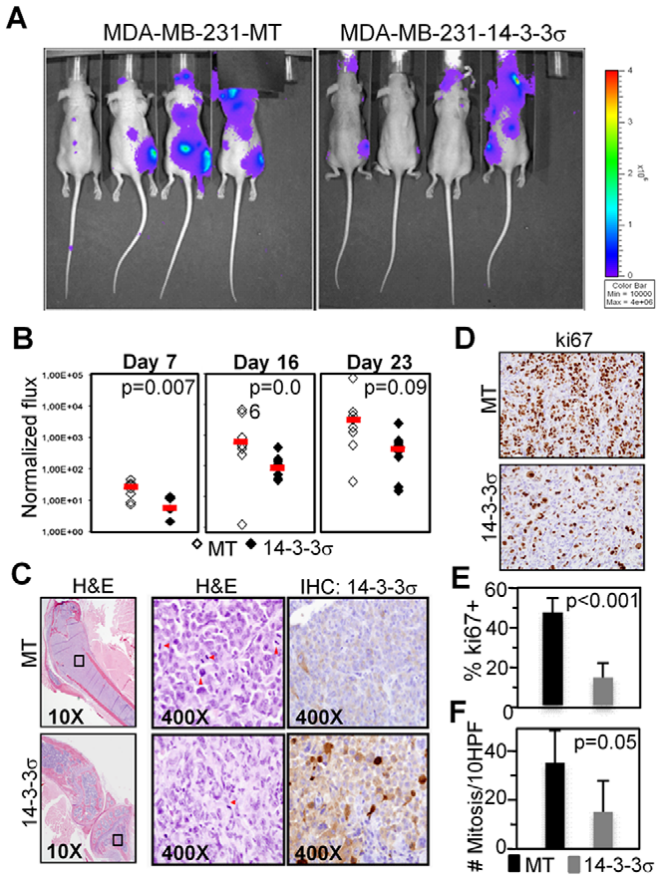
Re-expression of 14-3-3σ in MDA-MB-231 cells inhibits bone metastasis in vivo. (A) Representative bioluminescence images of mice injected with 0.5×10^6^ control or 14-3-3σ-overexpressing MDA-MB-231 cells 4 weeks after intracardiac injection. (B) Scattered plot of the dorsal photon flux (normalized to Day 0) at different times after injection. Each symbol represents one animal (C–D) H&E staining and IHC for 14-3-3σ (C) and IHC for ki-67 (D) of a representative bone-metastasis from a mouse injected with control (MT) or 14-3-3σ-expressing cells. (E–F) Quantification of cells expressing the proliferation marker ki-67 (E) and number of mitotic figures in the H&E staining (F) determined by counting 10 different high power fields per tumor (3 tumors counted for each group).

To investigate whether 14-3-3σ affects the metastatic breast cancer phenotype in vivo, we transduced control (MT) or 14-3-3σ-expressing MDA-MB-231 cells with a luciferase reporter construct and injected them intracardiacally in nude mice (8 animals per group). Then, we measured the total tumor load and their spatial distribution by life imaging at different time points after injection (Figure 5A and 5B). We confirmed that all animals contained comparable tumor cells at day one after injection, however one week post injection outgrowths arising in the animals inoculated with 14-3-3σ expressing cells were significantly smaller compared with those injected with control cells (p = 0.007)(Figure 5B). Most of the metastatic lesions were localized in the bones, specifically in the tibia and in the femur and were histologically comparable between the groups, displaying a poorly differentiated phenotype, nuclear pleomorphism and high mitotic rate (Figure 5C). Interestingly, control cells generated a higher number of bone metastasis (8/16 legs, 50%) than cells expressing 14-3-3σ (5/16 legs, 31%), and IHC analysis of the bones at week 4, when animals were sacrificed, demonstrated that control tumors were bigger (Figure 5C) and showed significantly higher numbers of proliferating cells (Figure 5D, 5E and 5F).

### Activation of NF-κB associates with absence of 14-3-3σ and poor prognosis in breast cancer patients

**Figure 6:**
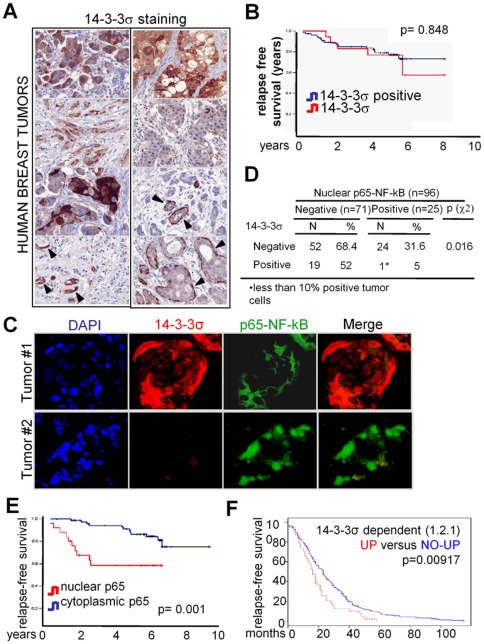
14-3-3σ-dependent NF-κB activity correlates with poor prognosis in breast cancer patients. (A) Representative tumors showing different patterns of 14-3-3σ expression, that was distributed both in cytoplasm and nucleus of tumor cells. Consistently, positive 14-3-3σ staining was observed in myoepithelial cells of normal ducts and ductal carcinoma *in situ* (arrowheads). (B and E) Kaplan-Meier curves depict cumulative disease-free survival of breast cancer patients stratified by the absence of 14-3-3σ (B) and by the presence of nuclear p65 (E). (C) Double IF of representative breast tumors showing that nuclear p65 distribution was restricted to the 14-3-3σ negative samples (D) Cross-tabulation of 14-3-3σ expression and NF-κB activation (as determined by nuclear p65) in the different tumors analyzed. (F) Kaplan-Meier curve depict relapse-free survival of breast cancer patients stratified by up-regulation of 14-3-3σ-dependent signature in the primary tumors.

Finally, we examined whether 14-3-3σ levels in chemo-naïve early human breast tumors correlated with clinical outcome and NF-κB activation. We found that about 80% of the tumors analyzed were negative for 14-3-3σ expression, which was detected at different levels in the normal glands of the same samples (Figure 6A). However, absence of 14-3-3σ did not correlate with clinical outcome in our series of 96 patients (Figure 6B). Notably, we detected nuclear p65 in a total of 25 tumor samples (26%), being 24 of them negative for 14-3-3σ (Figures 6C), indicating that NF-κB activation associates with loss of 14-3-3σ in these tumors (p = 0.016) (Figure 6D). Importantly, NF-κB activation in the tumors was associated with reduced disease-free survival in the univariate analysis of the patients (Hazard ratio -HR- (95%CI): 3.68 (1.56 to 8.60), p = 0.001) (Figure 6E and Table 1). In the multivariate Cox regression analysis we found that nuclear p65 was an independent predictor when adjusted by the rest of significant factors on the univariate testing. NF-κB activation showed the highest risk for relapse compared to the tumor grade or the presence of infiltrated lymph nodes (p<0.001, Hazard Ratio of 8.89, 6.68 and 6.15, respectively) (Table 1). Analysis of breast cancer experiments containing both microarray expression and clinical data demonstrated that patients with tumors over-expressing the 14-3-3σ-dependent signature showed a poorer relapse-free survival compared with the rest of the patients (p = 0.00917)(Figure 6F) (experiment GSE12276) [35]. Together our data indicates that pro-tumorigenic effects of 14-3-3σ deletion in breast cancer are associated with its role as an NF-κB regulator.

**Table 1:** Relapse-free survival analysis.

	Univariate (n = 96)			Multivariate (n = 96)		
	HR	95% CI	P	HR	95% CI	P
Variable						
Age			0.269			
Premenopausal	1.00			-		
Postmenopausal	0.44	0.10 to 1.89		-		
Tumor size, mm			0.020			0.167
≤20	1.00			1.00		
21–50	3.85	1.47 to 10.07		2.41	0.80 to 7.25	
>50	1.77	0.35 to 8.81		1.74	0,73 to 7.54	
Tumor grade			0.007			0.024
I	1.00			1.00		
II	2.29	0.48 to 10.86		1.76	0.34 to 9.25	
II	7.02	1.56 to 31.46		6.68	1.15 to 38.75	
Lymph nodes			0.006			0.007
None	1.00			1.00		
1–3	1.22	0.39 to 3.80		0.63	0.19 to 2.17	
4–9	0.38	0.49 to 2.93		1.33	0.30 to 3.06	
>9	5.64	1.96 to 16.22		6.16	1.49 to 25.39	
Histology			0.603			
Ductal	1.00			-		
Lobular	0.91	0.54 to 2.51		-		
Others	1.01	0.32 to 2.74		-		
Hormonal receptor status			0.002			0.402
Negative	1.00			1.00		
Positive	0.27	0.11 to 0.62		0.63	0.22 to 1.84	
HER2 status			0.062			
Negative	1.00			-		
Positive	1.89	0.94 to 5.59		-		
Proliferation (Ki67)			0.022			0.057
Low proliferation	1.00			1.00		
High proliferation	2.71	1,15 to 6.34		2.88	0.97 to 8.56	
Adjuvant chemotherapy			0.396			
No	1.00			-		
Yes	0.68	0.28 to 1.64		-		
Adjuvant hormonotherapy			0.151			
No	1.00			-		
Yes	0.54	0,23 to 1.26		-		
p65-NF-kB			0.001			<0.001
Cytoplasmic expression	1.00			1.00		
Nuclear expression	3.68	1.56 to 8.60		8.89	2.99 to 26.39	

Abreviations: DFS, disease free survival; HR, hazard ratio; CI, confidence interval; HER2, human epidermal growth factor receptor 2.

Relapse free survival analysis of the group of patients that were studied for the presence of nuclear p65. Commonly used clinical predictors such as tumor grade and size, or the number of infiltrated lymph nodes were studied in comparison with the status of p65.

## Discussion

We have identified 14-3-3σ as a negative regulator of NF-κB in breast cancer and demonstrated that loss of 14-3-3 expression is directly associated with the capacity of breast cancer cells to metastasize.

Chronic NF-κB activation has been involved in many types of cancer, mostly associated with immune cell infiltration, which express pro-inflammatory cytokines that activate the pathway in the epithelial cells [16], [36], [37], [38]. In agreement with this, most of the tumor-related effects of NF-κB activation, for example in liver and intestine, are generally attenuated by specific abrogation of the pathway in the immune cells [39], [40]. Similarly, NF-κB activity in breast cancer metastasis are likely induced by infiltrating T-cells [16] in contrast to the cell autonomous activation of NF-κB downstream of Src [41], Her2 [42] or by mutation of p53 [43]. The convergence of different oncogenic pathways on NF-κB opens the possibility to specifically target this transcription factor or other elements of the pathway for therapeutical intervention in breast cancer, a strategy that is currently tested in patients with cancer-related bone metastases using proteasome inhibitors (18454159) or antibodies against RANKL [PMID 21060033, 17785705, 19237632] ([44].

We previously demonstrated the involvement of 14-3-3β and 14-3-3η proteins in p65 nuclear export after TNFα activation in HEK-293T cells [27]. We now showed that 14-3-3σ plays a non-redundant function in mammary cells facilitating p65 nuclear export, although we cannot exclude other mechanisms that affects intracellular distribution of p65. In agreement with this, activation of NF-κB was specifically found in human tumors lacking 14-3-3σ. Deletion of 14-3-3σ has been consistently associated with breast cancer as it plays tumor suppressor functions in this [45] and other types of cancer [46], [47]. We have now demonstrated that absence of 14-3-3σ expression by itself does not correlate with poorer clinical outcome, but it is absolutely required for NF-κB activation, which is intimately related with patient relapse and clinical outcome. This finding indicates that despite the important functions of 14-3-3 as a regulator of CDC25, p53, FOXO, raf-1 or mTOR among others, modulation of the NF-κB pathway is the link between loss of 14-3-3σ and breast cancer patient outcome. In addition, we have identified a specific signature downstream of TNFα that depends on 14-3-3σ including previously characterized NF-κB-target genes such as IL6, IL8, GM-CSF or A20. The mechanisms underlying this gene specificity are still unknown but it could be related with the presence of specific histone modifications in the promoter of these genes [48]. Moreover, since TNFα activates other pathways such as JNK or the caspase cascade [49], we cannot exclude that 14-3-3σ-dependent signature includes some NF-κB-independent genes. In any case, 14-3-3σ-regulated genes affect the acquisition of specific capacities such as cell migration and bone metastasis in vivo, and its over-expression correlates with disease-free survival in breast cancer patients.

Considering the current availability of high throughput sequencing technology in clinical practice, our results are of critical importance for future stratification of breast cancer patients for personalized treatments.

## Materials and Methods

### Ethics Statement

Human tissue samples were obtained from the archive of The Pathology Department of Hospital del Mar with the approval of the Bank Tumor Committee, according to the Spanish Ethical regulations. This study was approved by the local Ethics Board (Clinical Research Ethical Committee of the Parc de Salut Mar, CEIC-IMAS2009/3515/) and followed the guidelines of the Declaration of Helsinki and patient identity of the pathological specimens remained anonymous in the context of this study. Informed written consent was obtained from all subjects.

All animal work has been performed at IRB, Parc Científic, Barcelona and followed the protocols approved by the Animal Care and Use Committee from Parc Científic de Barcelona. Decree 214/1997 regulates the use of animals for research. Along these lines the Autonomous Government of Catalonia approved the Animal Welfare Committee of the Parc Científic de Barcelona, responsible for evaluating activities undertaken in the centre.

### Cell lines and cultures

Seven human breast cancer cell lines (MDA-MB-231, MDA-MB-453, MDA-MB-468, SK-BR-3, BT-474, MCF-7 and T47D), a human cervical cancer cell line (HeLa) and a mammary epithelial one as control (MCF-10A) were purchased from the American Type Culture Collection and cultured at 37°C with 5% CO2 in DMEM (MDA-MB-231, MDA-MB-453, MDA-MB-468, BT-474, SK-BR-3, MCF-7, 2 supplemented with 10% fetal bovine serum. For BT-474, insulin (0.01 mg/ml) was added. Line MCF-10A was cultured in MEMB Basal Medium (Clonetics, cat. No. CC-3151) supplemented with MEGM Singlequots (Clonetics, cat. No. CC-4136): 2 ml BPE, 500 ng/ml hydrocortisone, 10 ng/ml hEGF and 5 mg/ml insulin. Cell lines were transfected using PPEI (PolySciences).

### Antibodies and Reagents

Anti p65 (sc-109 and sc-372), p50 (sc-7178), IκBα (sc-371), PAN-14-3-3 (sc-629), 14-3-3β (sc-17288) and 14-3-3σ (sc-7681 and sc-7683) were from Santa Cruz. α-α-tubulin was from Sigma, and α-ki67 from Novocastra. Secondary HRP-conjugated antibodies were from DAKO and fluorescein-conjugated secondary antibodies from Molecular Probes. BAY11-7082 (Calbiochem) was used at 100 µM and BAY65-5811 is a gift from Bayer. TNFα from Preprotech was used at 40 ng/ml.

### Pull-down Assays

PD assays were performed as previously described [50], Briefly, GST fusion proteins were purified and incubated with cell lysates for 45 min in a rotary shaker at 4°C. Precipitates were washed and analyzed by western blot.

### Subcellular fractionation and western blotting

For cytoplasm-nuclear separations, cells were lysed in 10 mM HEPES, 1.5 mM MgCl2, 10 mM KCl, 0.05%NP40 at pH 7.9, 10 min on ice and centrifuged at 3.000 rpm. Supernatants were recovered as cytoplasmic fraction and the pellets lysed in 5 mM HEPES, 1.5 mM MgCl2, 0.2 mM EDTA, 0.5 mM DTT and 26% glycerol and sonicated 5 min three times to recover the soluble nuclear fractions. Protein lysates were run in SDS/PAGE and transferred to immobilon-P transfer membranes (Millipore). Membranes were blocked with 4% dehydrated milk and blotted with the indicated antibodies O/N at 4°C. Secondary HRP-linked secondary antibodies were developed using the ECL system from Amersham.

### Coimmunoprecipitation assays

Precipitations were performed in 500 µl of PBS containing 0.5% Triton X-100, 1 mM EDTA, 100 µM sodium orthovanadate, 0.2 mM PMSF, 20 mM β-glycerophosphate and complete protease inhibitor cocktail (Roche). Lysates were incubated for 2 hours at 4°C with primary antibodies coupled to Protein A-Sepharose beads, washed and analyzed by western blot.

### Immunofluorescence

Cells were fixed in 4% paraformaldehyde and permeabilized with 0.3% Triton X-100 and 4% non-fat dry milk in PBS. Primary antibodies were incubated overnight and secondary antibodies for 90 min. Slides were mounted in Vectashield with DAPI (Vector) and visualized in an Olympus BX61. Images were analyzed using the Cell̂B Software from Olympus.

### Immunohistochemistry (IHC)

Paraffin-embedded 4 µm sections were kept one hour at 60°C and re-hydrated. Antigen retrieval was done in citrate buffer (pH 6.0) at 80°C, and primary antibodies were incubated overnight at 4°C. Samples were washed in ChemMate buffer solution and developed with Envision signal detection system (DAKO). Two investigators, blinded to clinical data, scored each sample, for nuclear p65 and 14-3-3σ staining. Concordance between investigators was higher than 95%.

### Electrophoretic mobility shift assay (EMSA)

Nuclear extracts were prepared as described [51]. Briefly, 3 µg of the indicated nuclear extracts were incubated with labeled double-stranded κB-binding consensus oligonucleotide (5-AGTTGAGGGGACTTTCCCAGGC-3) 15 min at room temperature in a final volume of 20 µL. Samples were run in 0.5% tris–borate–EDTA buffer on non-denaturating 4% polyacrylamide gels, which were vacuum-dried and subjected to autoradiography. Densitometric analysis of the bands was performed using the Adobe Photoshop CS4 software.

### qRT-PCR

Total RNA was isolated using RNeasy kit (Qiagen) and cDNA was obtained with RT-First Strand cDNA Synthesis kit (Amersham). qRT-PCR was performed in LightCycler 480 system sing SYBR Green I master kit (Roche).

### Microarray experiment

Control or 14-3-3σ-reconstituted MCF7 cells were incubated with TNFa for 0, 20 and 90 min in two independent experiments. RNA was obtained and RNA integrity assessed using Agilent 2100 Bioanalyzer (Agilent Technologies, Palo Alto, CA). All samples had an RNA integrity number (RIN)≥8.5. Amplification, labeling and hybridizations were performed according to protocols from Ambion and Affymetrix. Briefly, 250 ng of total RNA were amplified using the Ambion® WT Expression Kit (Ambion/Applied Biosystems, Foster City, CA, USA), labeled using the WT Terminal Labeling Kit (Affymetrix Inc., Santa Clara, CA, USA), and then hybridized to Human Gene 1.0 ST Array (Affymetrix) in a GeneChip® Hybridization Oven 640. Washing and scanning were performed using the Hybridization Wash and Stain Kit and the GeneChip® System of Affymetrix (GeneChip® Fluidics Station 450 and GeneChip® Scanner 3000 7G).

### Microarray data processing

Microarray data was processed with R statistical framework [52]. Aroma.affymetrix [53], corpcor [54] and Limma [55] packages were used to preprocess, batch adjust and determine the top 220 most differentially expressed genes across conditions with an adjusted p-value (False Discovery Rate, FDR) <0.05 [56]. The analysis was designed to identify genes that were induced by TNFα in control cells but not in 14-3-3σ-expressing MCF7 cells (group 1); genes induced in both control and 14-3-3σ-expressing MCF7 cells (group 2) including genes that were differentially induced in control and 14-3-3σ-expressing MCF7 cells (group 2.1). Groups 1 and 2.1 were included as 1_2.1 in subsequent analysis. Values represented for each probe set correspond to standard deviations after mean-centering the values in each row. Hierarchical clustering was performed on samples and also on the 220 most differentially expressed genes using Pearson correlation as distance measure and the average method, and the obtained clusters were used to generate the groups of genes for further analysis.

### Functional enrichment analysis of 14-3-3σ gene signatures

We performed enrichment analysis (EA) of the different groups (see microarray data processing) with Gene Ontology terms. This analysis reveals the enrichment of a particular functional term in a group of genes. The EA was done with binomial test using Gitools [57], a statistical framework for the analysis and visualization of genomics data. The annotation of genes to corresponding Gene Ontology Biological Process, Cell Location and Molecular Function terms were downloaded from Ensembl version 60 [58] using Gitools importer. The p-values resulting from the binomial test were adjusted for multiple testing using the Benjamini and Hochberg's method of FDR and we set the cut-off for significance at a 0.05 of the corrected p-value. Results are represented in a heat-map of p-values where colors towards red indicate significance and grey indicates no significant enrichment.

### 14.3.3σ dependent signature up-regulation in cancer gene modules

To test whether genes in the 14-3-3σ-dependent signature (group 1_2.1) are enriched in cancer samples, we used sets of genes significantly upregulated in different cancer genomic experiments as obtained from IntOGen [34]. Briefly, IntOGen contains a collection of independent cancer genomic datasets from different tumor types, which are processed to identify genes altered in a significant number of samples. Enrichment analysis was performed with Gitools as explained above.

### Relapse-free survival analysis

To test whether expression of genes in the 14-3-3σ dependent signature correlates with tumor relapse we used Sample Level Enrichment Analysis (SLEA) as described in Gundem and Lopez-Bigas 2012 [53] with a dataset containing 204 primary tumors from breast cancer patients with known site of relapse (GEO id GSE12276, [35]. Briefly, SLEA assesses the transcriptional status of a set of genes in each sample using Z-score test (see [53] for details), the result is a Z-score per sample indicating the transcriptional status of the gene set in each sample. High Z-scores indicate that the set of genes tend to have high expression, low Z-scores indicate that the set of genes tend to have low expression and Z-scores around 0 indicate that they do not show a clear tendency to be misregulated in any direction. We used the Z-score to stratify the 204 samples according to the expression levels of genes in 14-3-3σ dependent signature and generated two groups: samples with significant up-regulation of 14-3-3σ dependent signature (n = 38) and the rest of samples (n = 166). Analysis of the relapse-free survival of patients in the two groups was performed using R Bioconductor survival package [52], [59].

### Metastasis Assay in nude mice

A cell suspension of 0.5×10^6^ MDA-MB-231 breast cancer cells was injected in the left cardiac ventricle of anesthetized 6–7-week-old nude mice. Tumor development was monitored by weekly bioluminescence imaging using the IVIS-200 imaging system from Xenogen [60]. Bone metastatic lesions were localized by ex vivo bioluminescence imaging and confirmed by histological analysis after necropsy. Bones were decalcified, fixed and processed for histological analysis (Hematoxylin & Eosin staining, H&E) and IHC. All animal work followed the protocols approved by the Animal Care and Use Committee of the Barcelona Science Parc.

### Computational promoter analysis

Presence of NF-κB binding sites in the promoters of interest (from −3000 bp to +100 bp to the transcription start site (TSS)) was predicted using the Genomatix software.

## Supporting Information

Figure S1 **Immunofluorescence with specific α-p65-NF-κB antibody (sc-109) of wildtype and p65-deficient mouse embryonic fibroblasts incubated with TNFα at the indicated times.**
(TIF)

Figure S2 **(A, B) Western blot analysis of IκBα expression in the indicated cell lines untreated or treated with TNFα.**
(TIF)

Figure S3 **(A) Immunofluorescence with specific α-p65-NF-κB antibody of MDA-MB-231 control or 14-3-3σ-expressing (clone#34) cells incubated with TNFα at the indicated times.** (B) Quantification of p65-NF-κB subcellular distribution determined by Immunofluorescence in one representative of three independent experiments: cytoplasmic: homogenous or cytoplasmic staining and nuclear: preferentially nuclear distribution. (C) Western blot analysis of 14-3-3σ expression in different cell clones.
(TIF)

Figure S4 **(A) Immunofluorescence (upper panel) and western blot analysis (lower panel) of 14-3-3σ expression in MCF7 cells carrying a doxycycline-inducible construct, untreated or treated with doxycycline for 16 h.** (B) Quantification of p65-NF-κB subcellular distribution in these cells in one representative experiments (from three independent experiments): cytoplasmic: homogenous or cytoplasmic staining and nuclear: preferentially nuclear distribution.
(TIF)

Figure S5 **List of genes included in groups 1 and 2.1.**
(TIF)

Figure S6 **Quantitative PCR analysis of MCF7 cells carrying a doxycycline-inducible 14-3-3σ construct to confirm the effects in the TNFα-dependent expression of randomly selected genes from 1.2.1 group.**
(TIF)

Figure S7 **Quantitative PCR analysis of randomly selected genes from 1.2.1 group in control or 14-3-3σ-expressing MDA-MB-231 cells.**
(TIF)

Figure S8 **Analysis of gene promoters using the Genomatix software to determine the presence of NF-κB binding sites in the selected 14-3-3σ-dependent genes.**
(TIF)

Figure S9 **Cell-cycle profiles (A) and apoptotic ratios as determined by AnexinV-binding (B) on control and 14-3-3σ-expressing MCF7 cells.**
(TIF)

Figure S10 **Cell-cycle profiles (A) and apoptotic ratios as determined by AnexinV-binding (B) on control and different 14-3-3σ-expressing MDA-MB-231 cell clones.**
(TIF)

Figure S11 **Wound-healing assay using control or 14-3-3σ-expressing MDA-MB-231 cells. Specific IKK inhibitor BAY11-7082 was used to inhibit NF-κB activity.**
(TIF)

Figure S12 **Representative wound-healing assay using MDA-MB-231 and MCF7 cells untreated or treated with the IKK inhibitor BAY11-7082 or with TNFα at the beginning of the experiment.** Immunofluorescence with α-p65-NF-κB antibody showing nuclear p65-NF-κB translocation in the migrating cells (right panel).
(TIF)

Figure S13 **Effects of blocking NF-κB activity with two different IKK inhibitors (BAY11-7082 and BAY65-5811) on the migratory capacity of MDA-MB-231 cells in transwell experiments.**
(TIF)

Figure S14 **Wound-healing assay using control MCF10A cells or cells transduced with different shRNA against 14-3-3σ.**
(TIF)
