# Supplementary figures and images for: Inhibition of Specific NF-κB Activity Contributes to the Tumor Suppressor Function of 14-3-3σ in Breast Cancer
Source: PLoS One. 2012 May 31;7(5):e38347. doi: 10.1371/journal.pone.0038347 (PMC3364992; doi:10.1371/journal.pone.0038347)

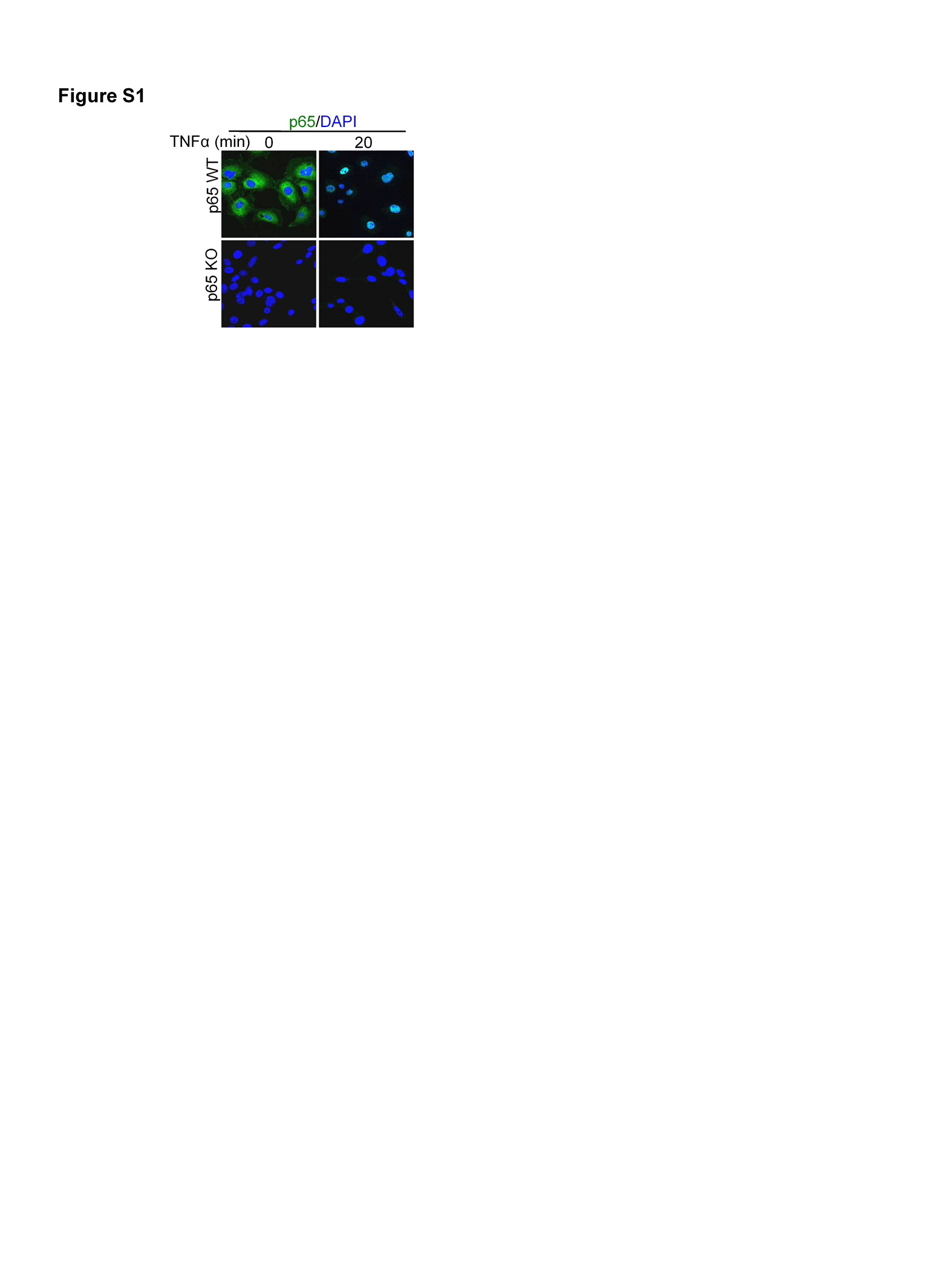

Supplement: Figure S1 — Immunofluorescence with specific α-p65-NF-κB antibody (sc-109) of wildtype and p65-deficient mouse embryonic fibroblasts incubated with TNFα at the indicated times. (TIF) [file pone.0038347.s001.tif]

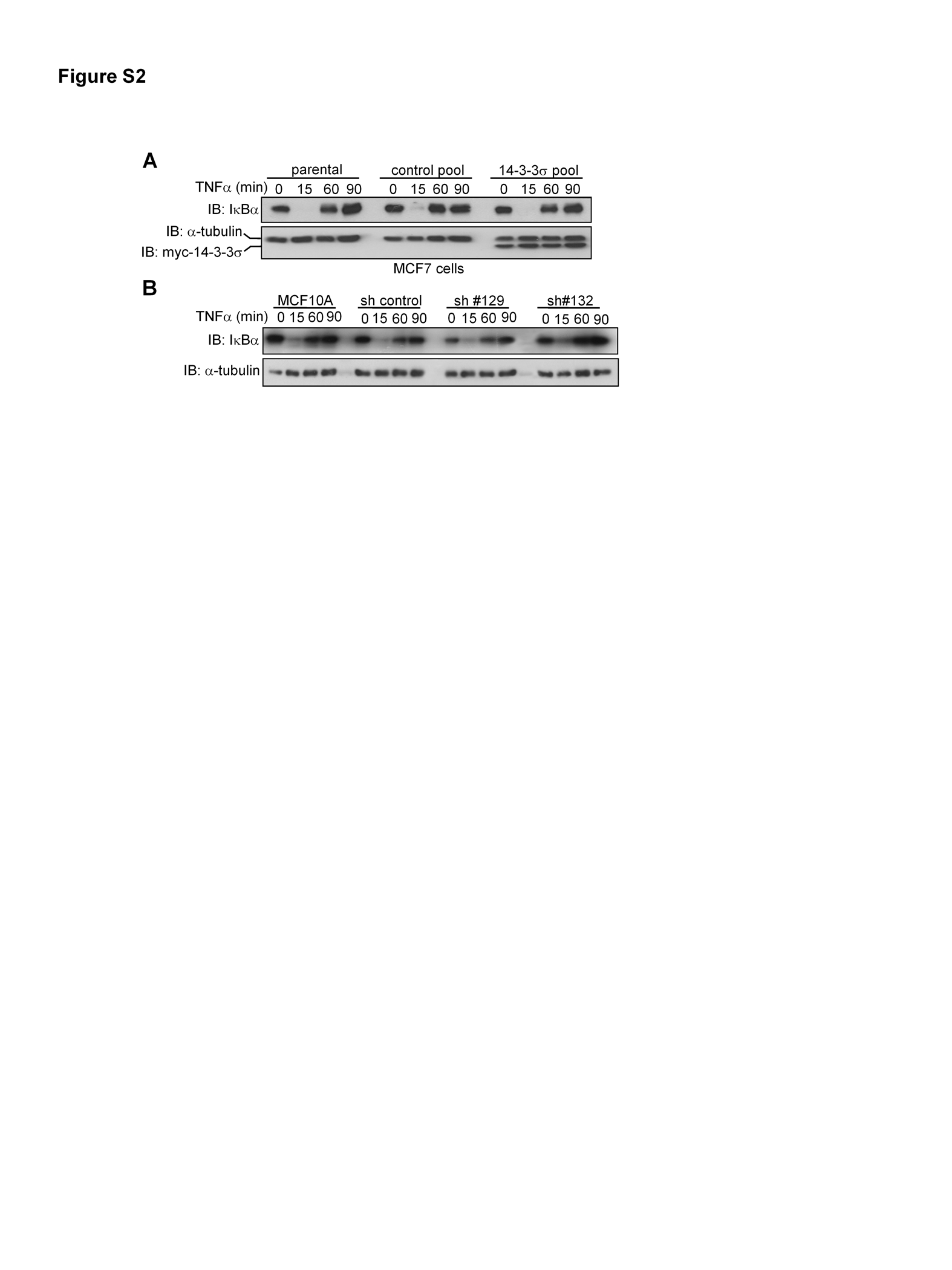

Supplement: Figure S2 — (A, B) Western blot analysis of IκBα expression in the indicated cell lines untreated or treated with TNFα. (TIF) [file pone.0038347.s002.tif]

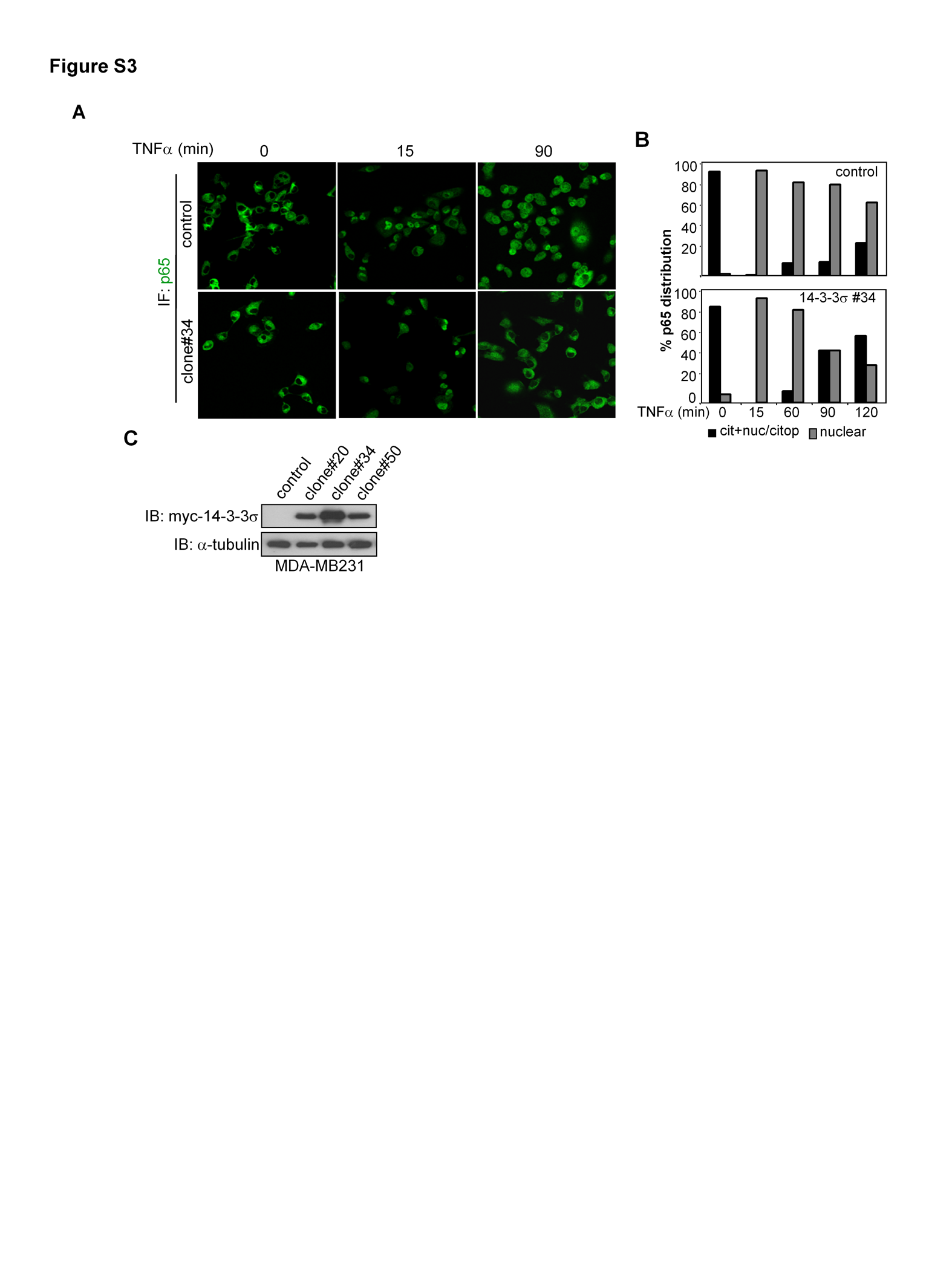

Supplement: Figure S3 — (A) Immunofluorescence with specific α-p65-NF-κB antibody of MDA-MB-231 control or 14-3-3σ-expressing (clone#34) cells incubated with TNFα at the indicated times. (B) Quantification of p65-NF-κB subcellular distribution determined by Immunofluorescence in one representative of three independent experiments: cytoplasmic: homogenous or cytoplasmic staining and nuclear: preferentially nuclear distribution. (C) Western blot analysis of 14-3-3σ expression in different cell clones. (TIF) [file pone.0038347.s003.tif]

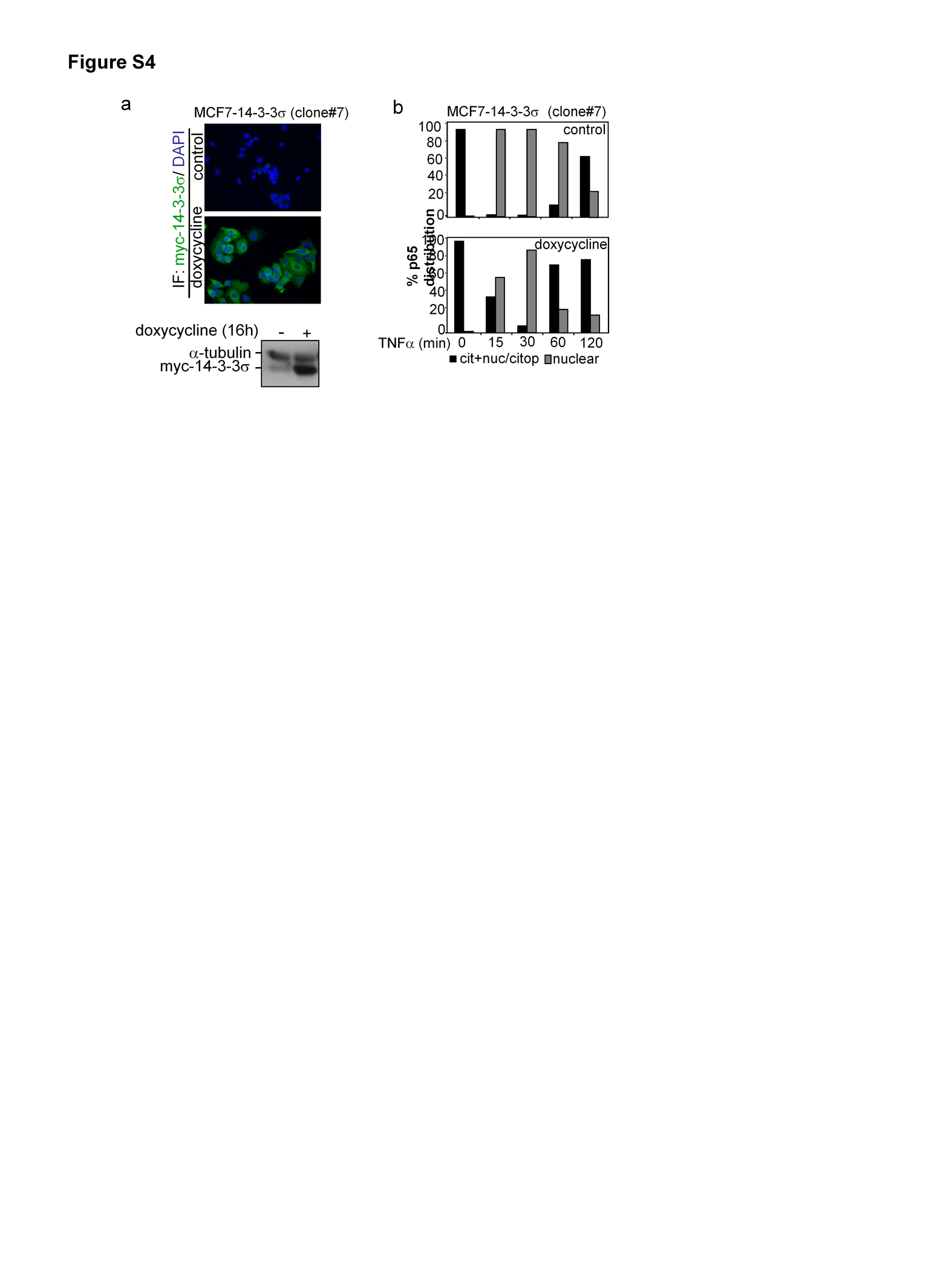

Supplement: Figure S4 — (A) Immunofluorescence (upper panel) and western blot analysis (lower panel) of 14-3-3σ expression in MCF7 cells carrying a doxycycline-inducible construct, untreated or treated with doxycycline for 16 h. (B) Quantification of p65-NF-κB subcellular distribution in these cells in one representative experiments (from three independent experiments): cytoplasmic: homogenous or cytoplasmic staining and nuclear: preferentially nuclear distribution. (TIF) [file pone.0038347.s004.tif]

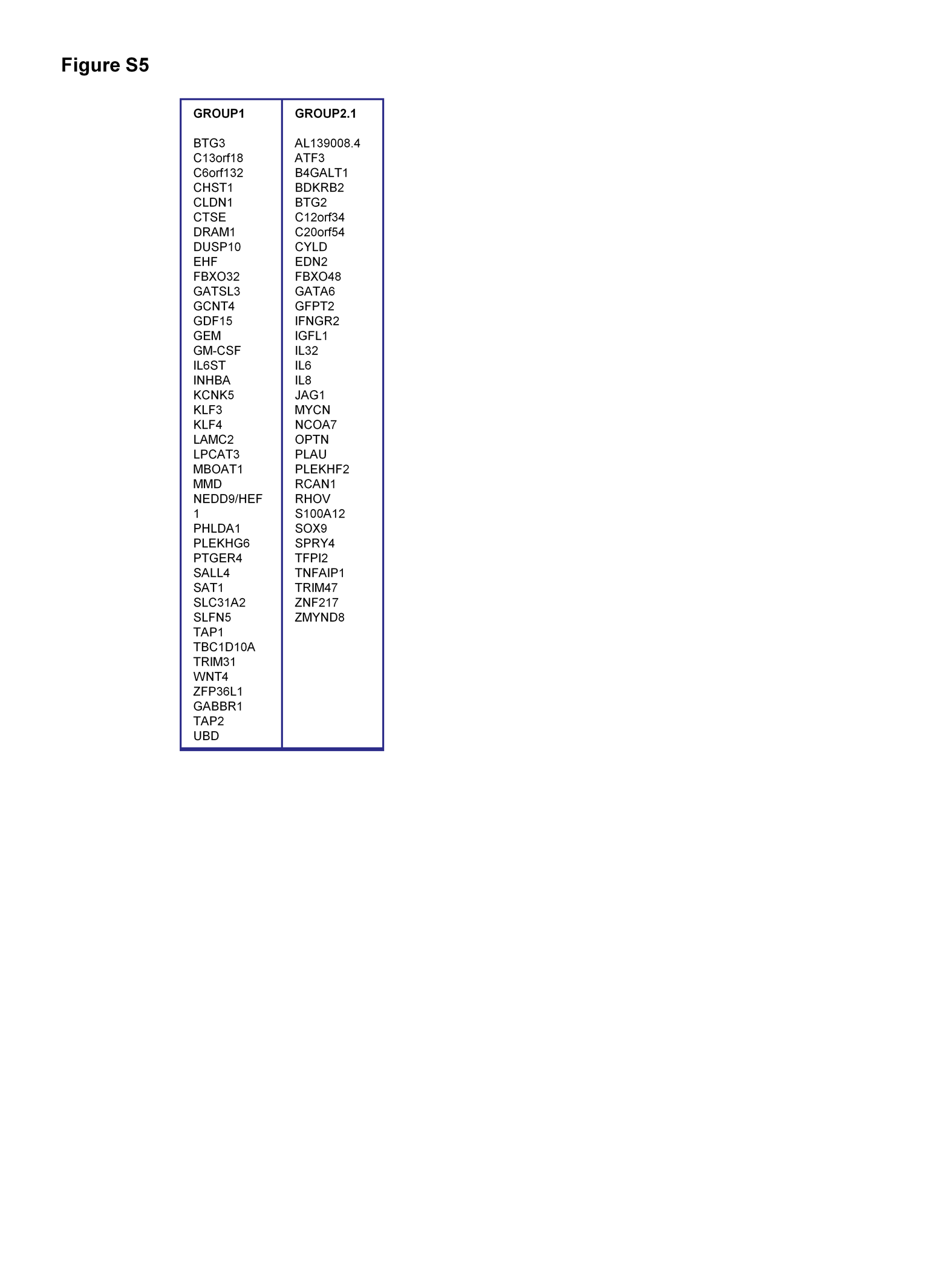

Supplement: Figure S5 — List of genes included in groups 1 and 2.1. (TIF) [file pone.0038347.s005.tif]

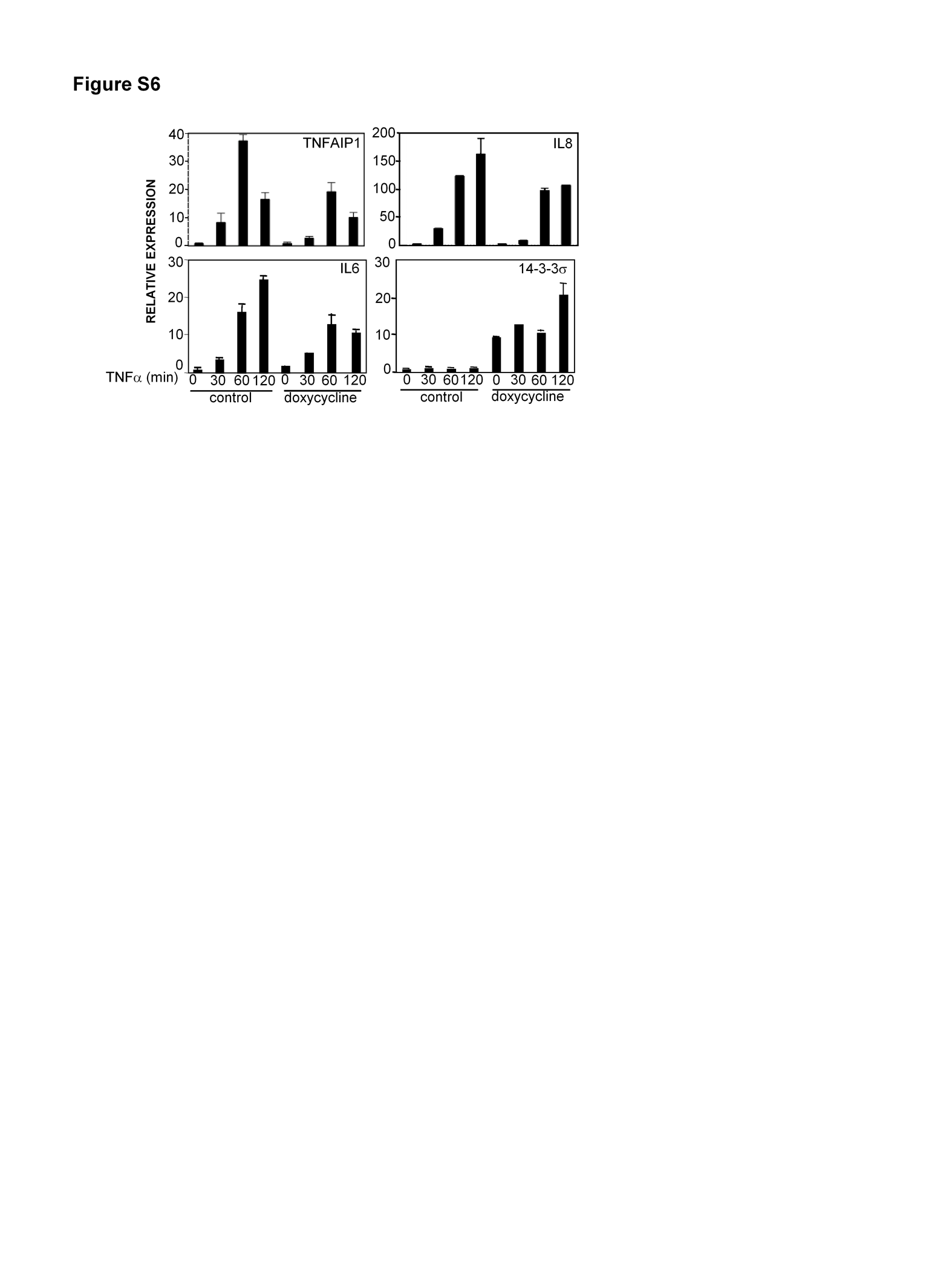

Supplement: Figure S6 — Quantitative PCR analysis of MCF7 cells carrying a doxycycline-inducible 14-3-3σ construct to confirm the effects in the TNFα-dependent expression of randomly selected genes from 1.2.1 group. (TIF) [file pone.0038347.s006.tif]

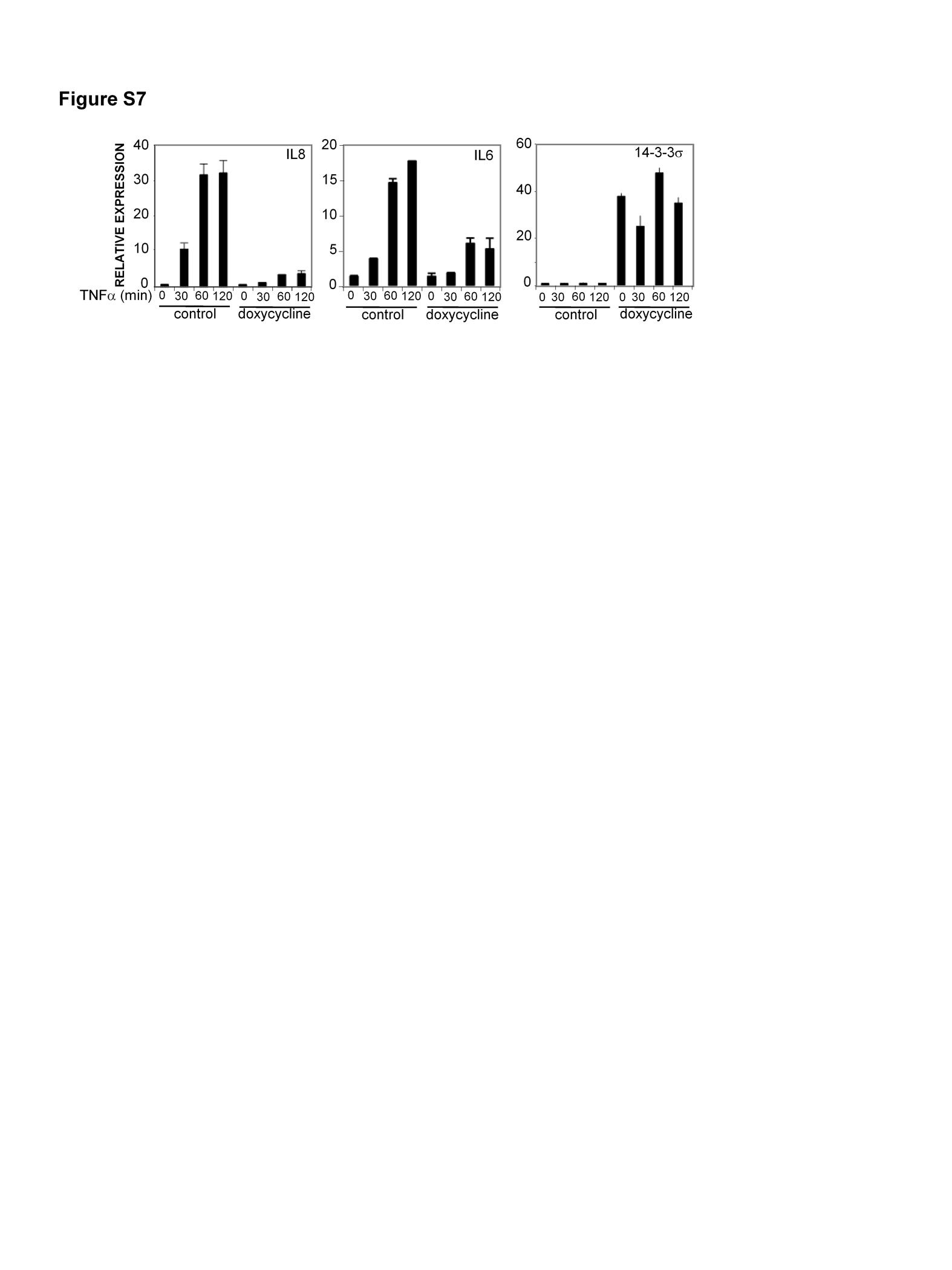

Supplement: Figure S7 — Quantitative PCR analysis of randomly selected genes from 1.2.1 group in control or 14-3-3σ-expressing MDA-MB-231 cells. (TIF) [file pone.0038347.s007.tif]

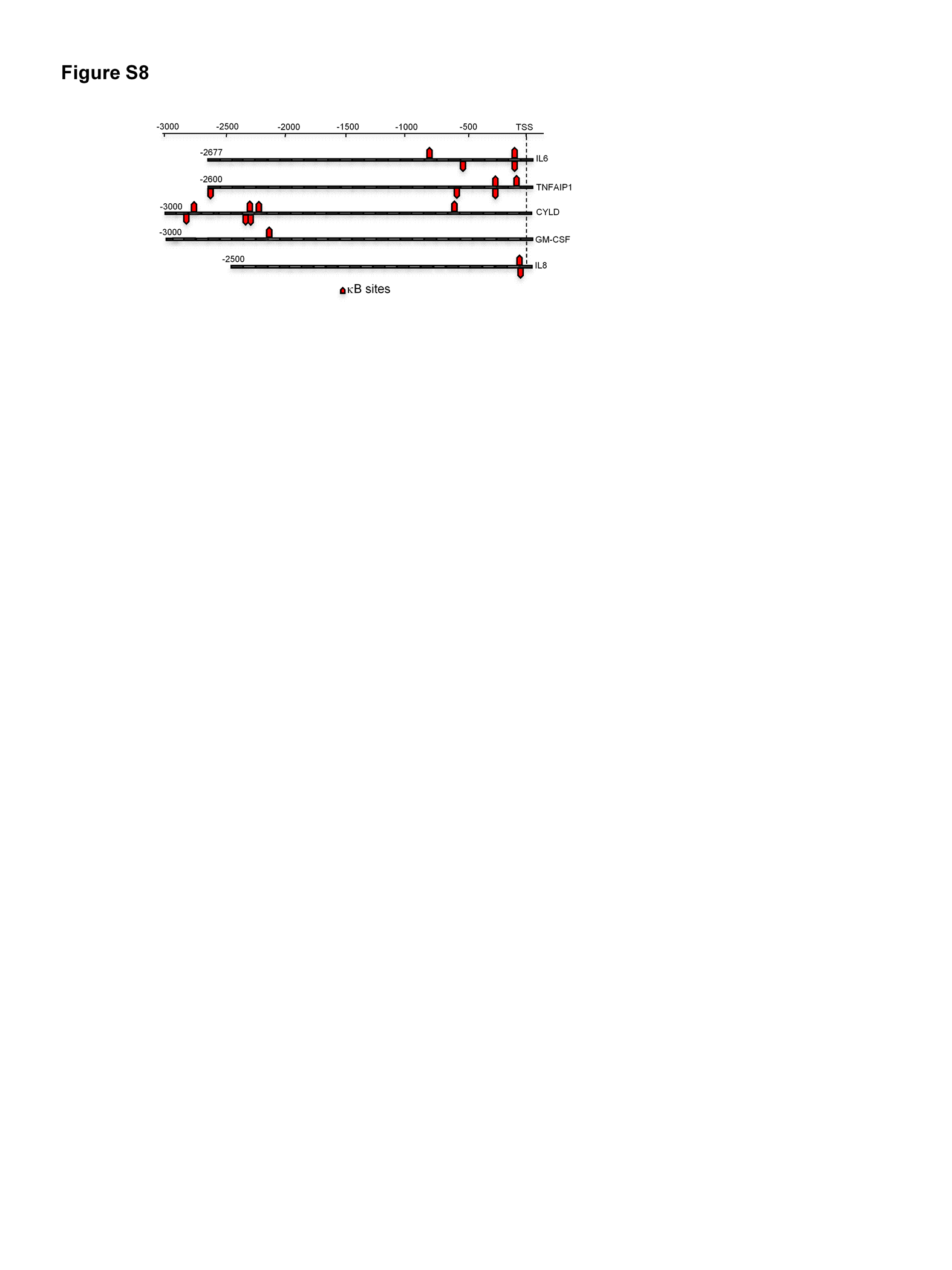

Supplement: Figure S8 — Analysis of gene promoters using the Genomatix software to determine the presence of NF-κB binding sites in the selected 14-3-3σ-dependent genes. (TIF) [file pone.0038347.s008.tif]

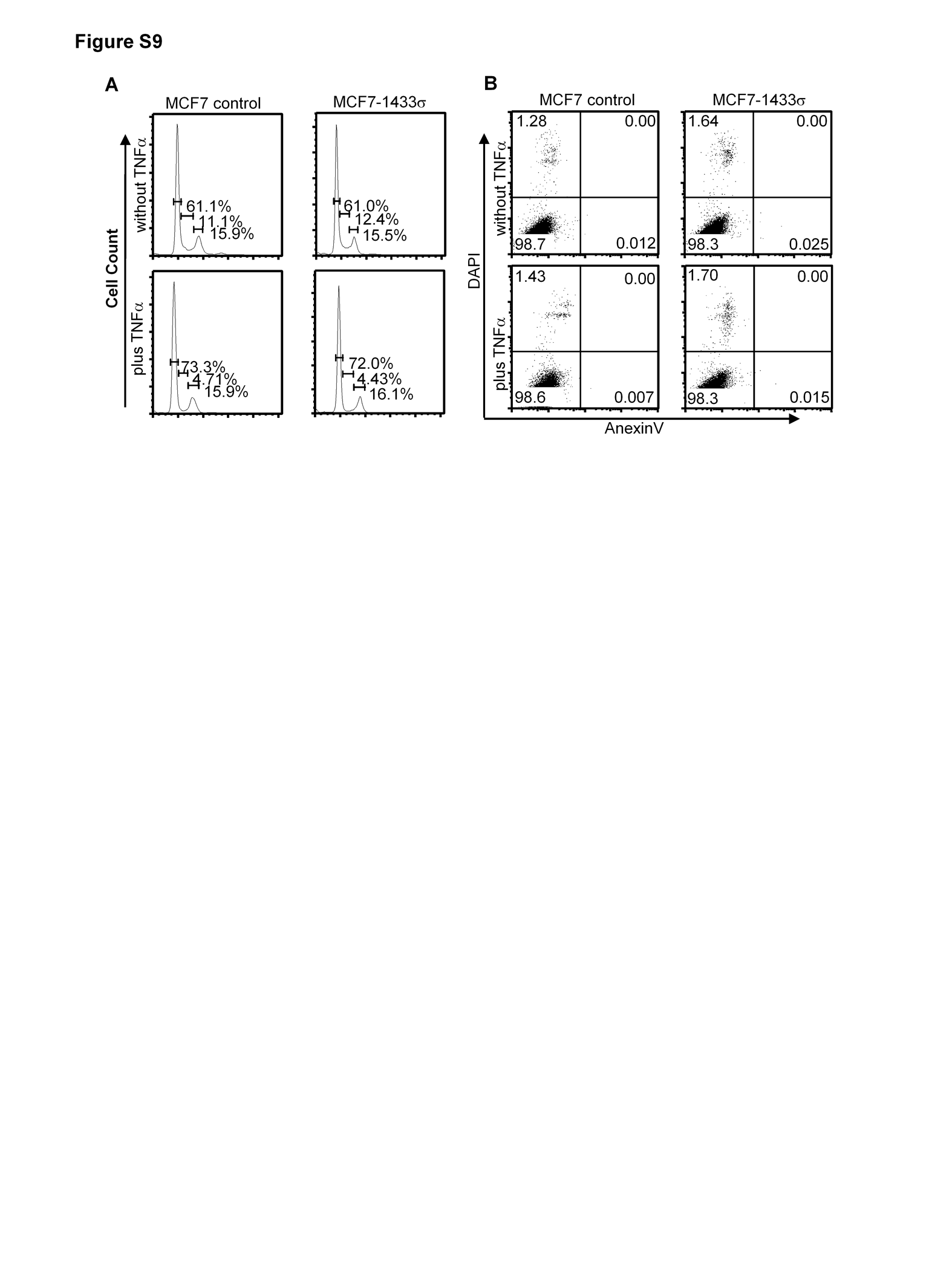

Supplement: Figure S9 — Cell-cycle profiles (A) and apoptotic ratios as determined by AnexinV-binding (B) on control and 14-3-3σ-expressing MCF7 cells. (TIF) [file pone.0038347.s009.tif]

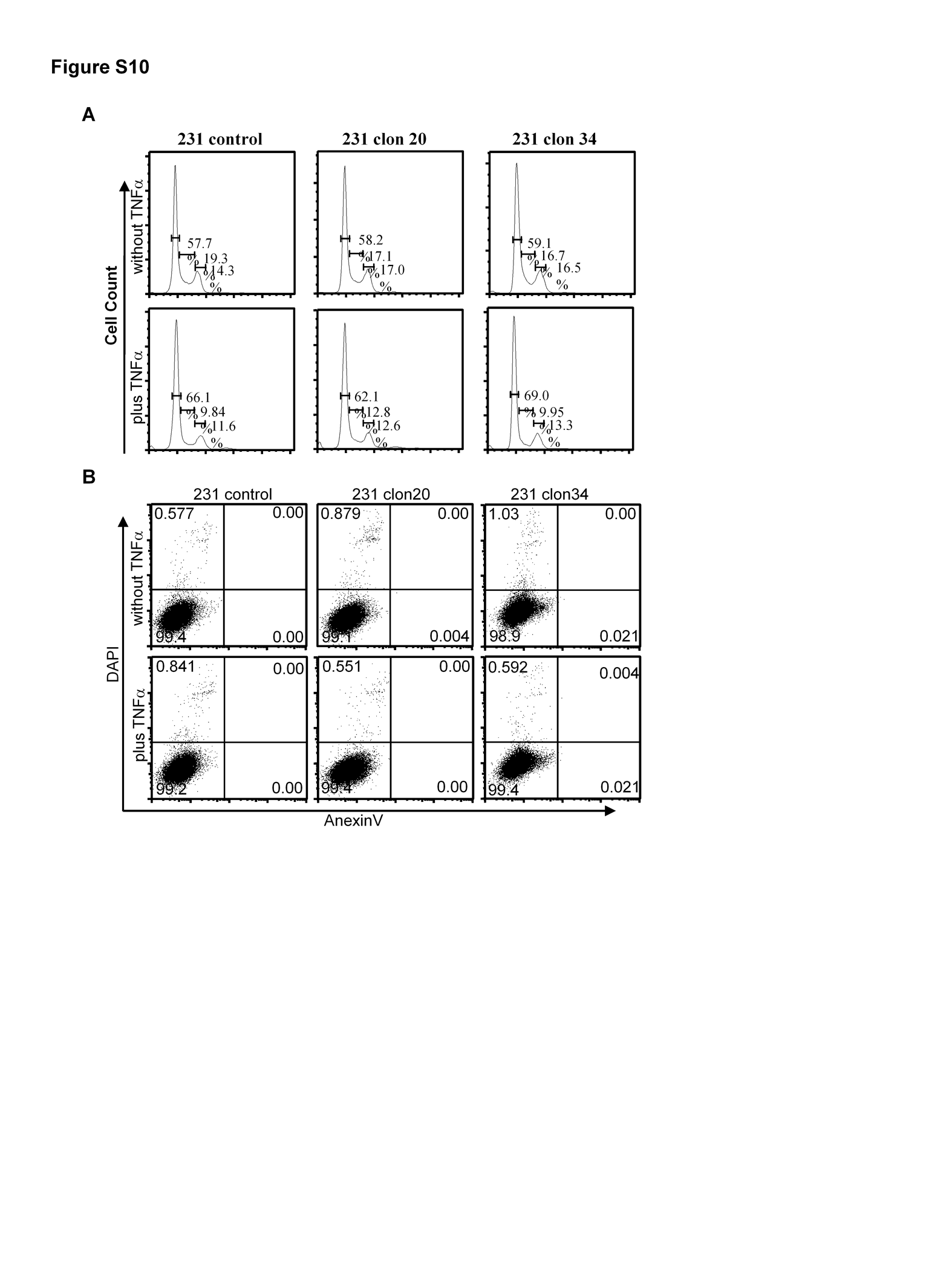

Supplement: Figure S10 — Cell-cycle profiles (A) and apoptotic ratios as determined by AnexinV-binding (B) on control and different 14-3-3σ-expressing MDA-MB-231 cell clones. (TIF) [file pone.0038347.s010.tif]

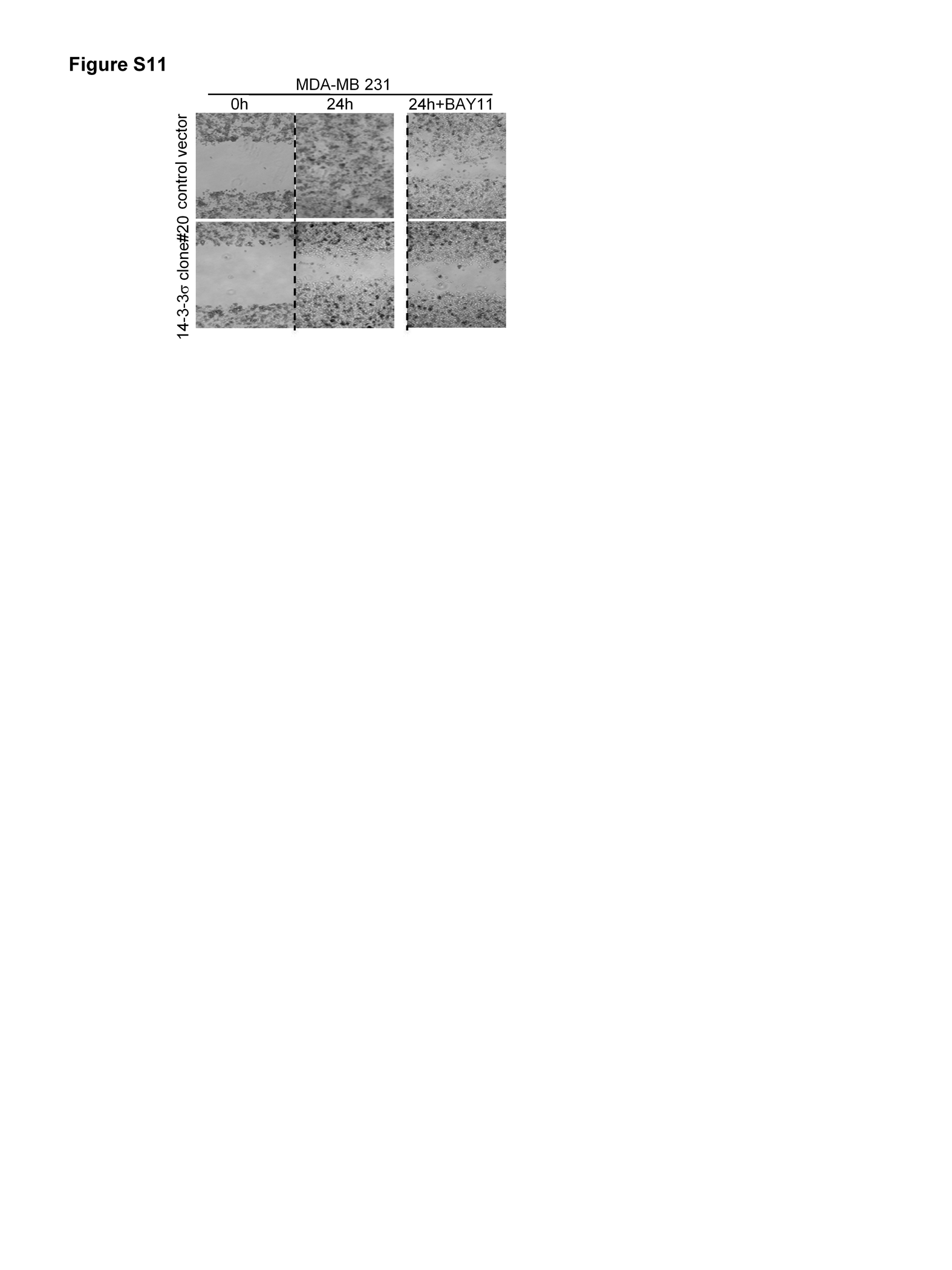

Supplement: Figure S11 — Wound-healing assay using control or 14-3-3σ-expressing MDA-MB-231 cells. Specific IKK inhibitor BAY11-7082 was used to inhibit NF-κB activity. (TIF) [file pone.0038347.s011.tif]

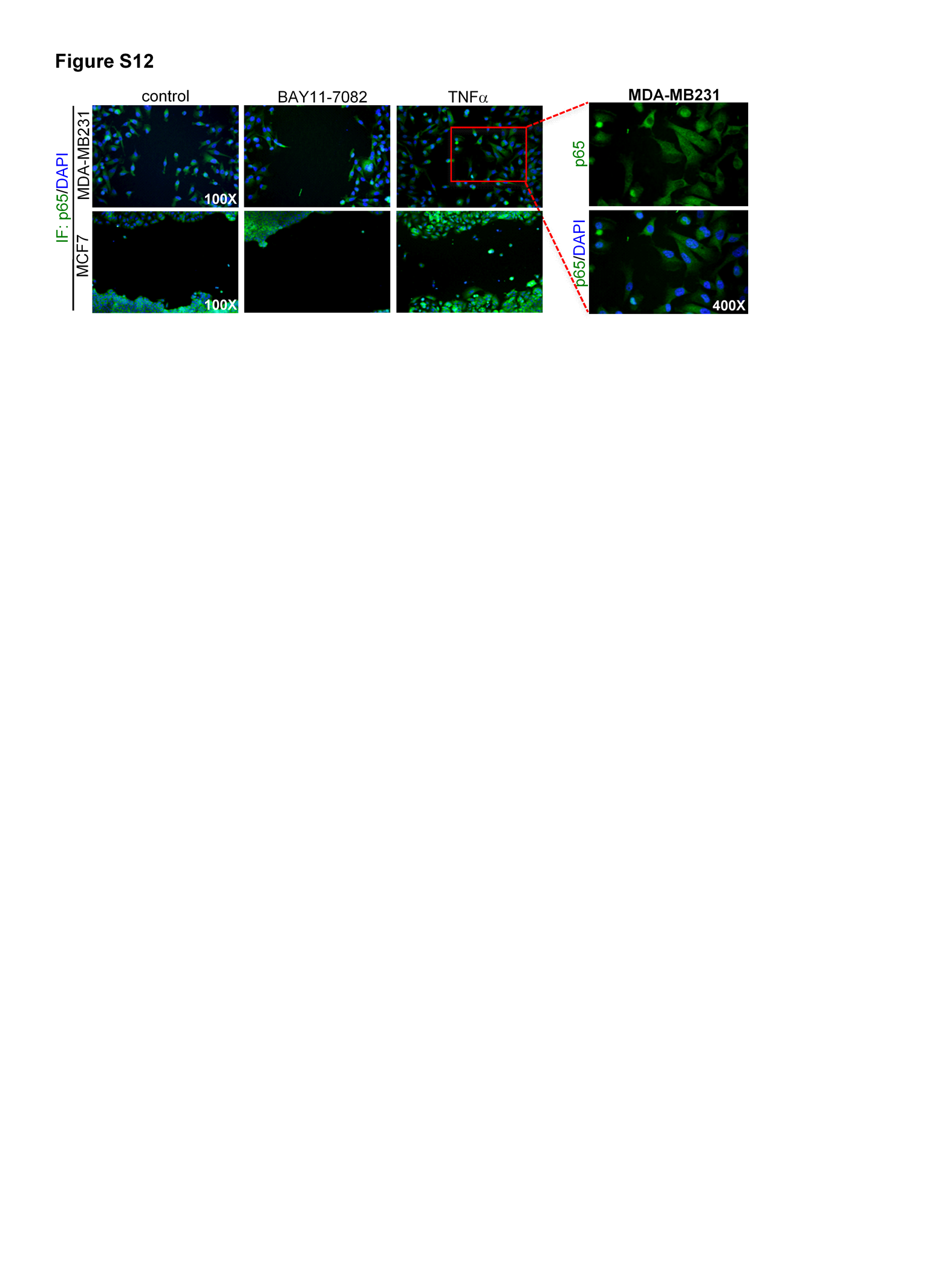

Supplement: Figure S12 — Representative wound-healing assay using MDA-MB-231 and MCF7 cells untreated or treated with the IKK inhibitor BAY11-7082 or with TNFα at the beginning of the experiment. Immunofluorescence with α-p65-NF-κB antibody showing nuclear p65-NF-κB translocation in the migrating cells (right panel). (TIF) [file pone.0038347.s012.tif]

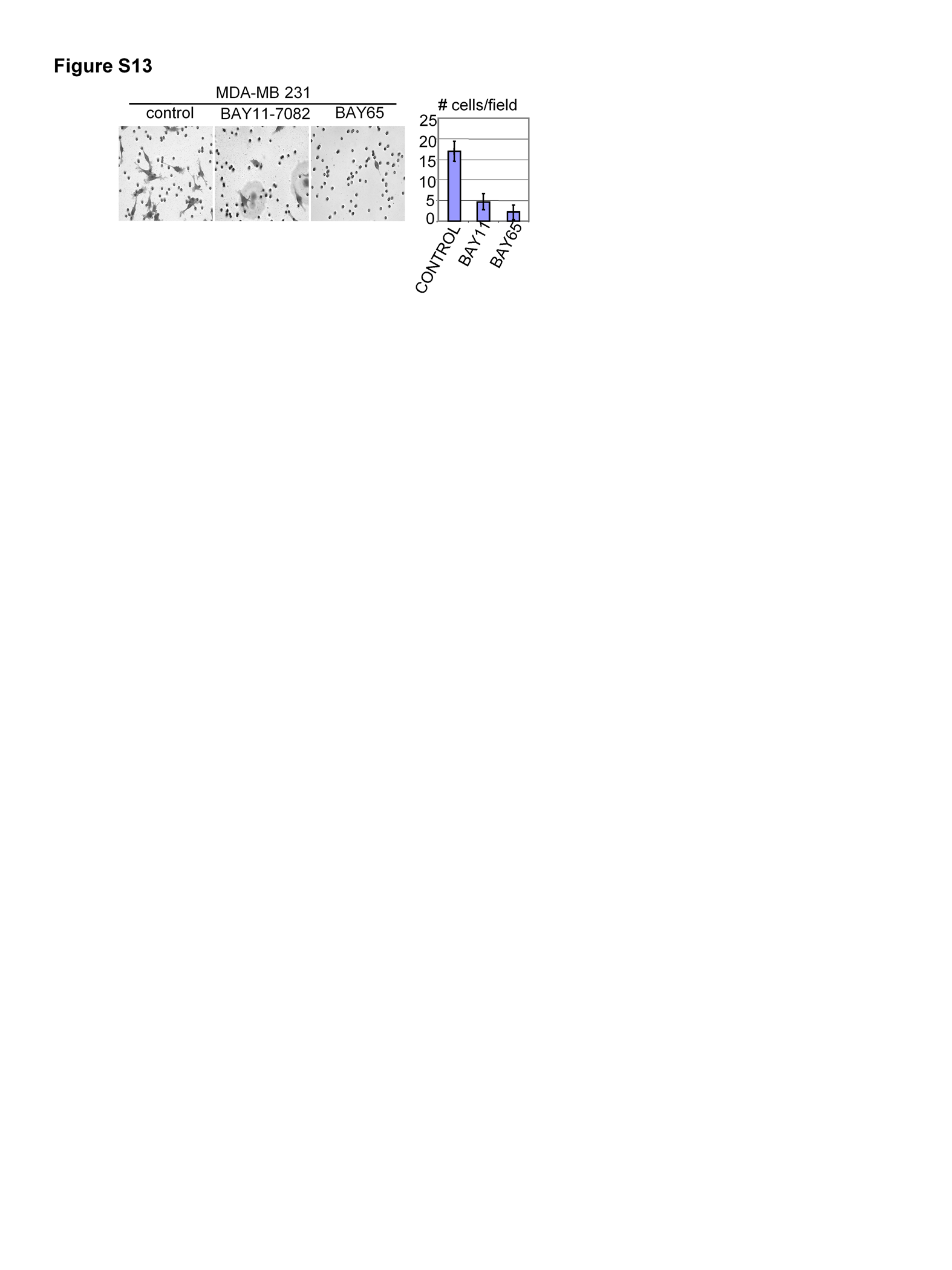

Supplement: Figure S13 — Effects of blocking NF-κB activity with two different IKK inhibitors (BAY11-7082 and BAY65-5811) on the migratory capacity of MDA-MB-231 cells in transwell experiments. (TIF) [file pone.0038347.s013.tif]

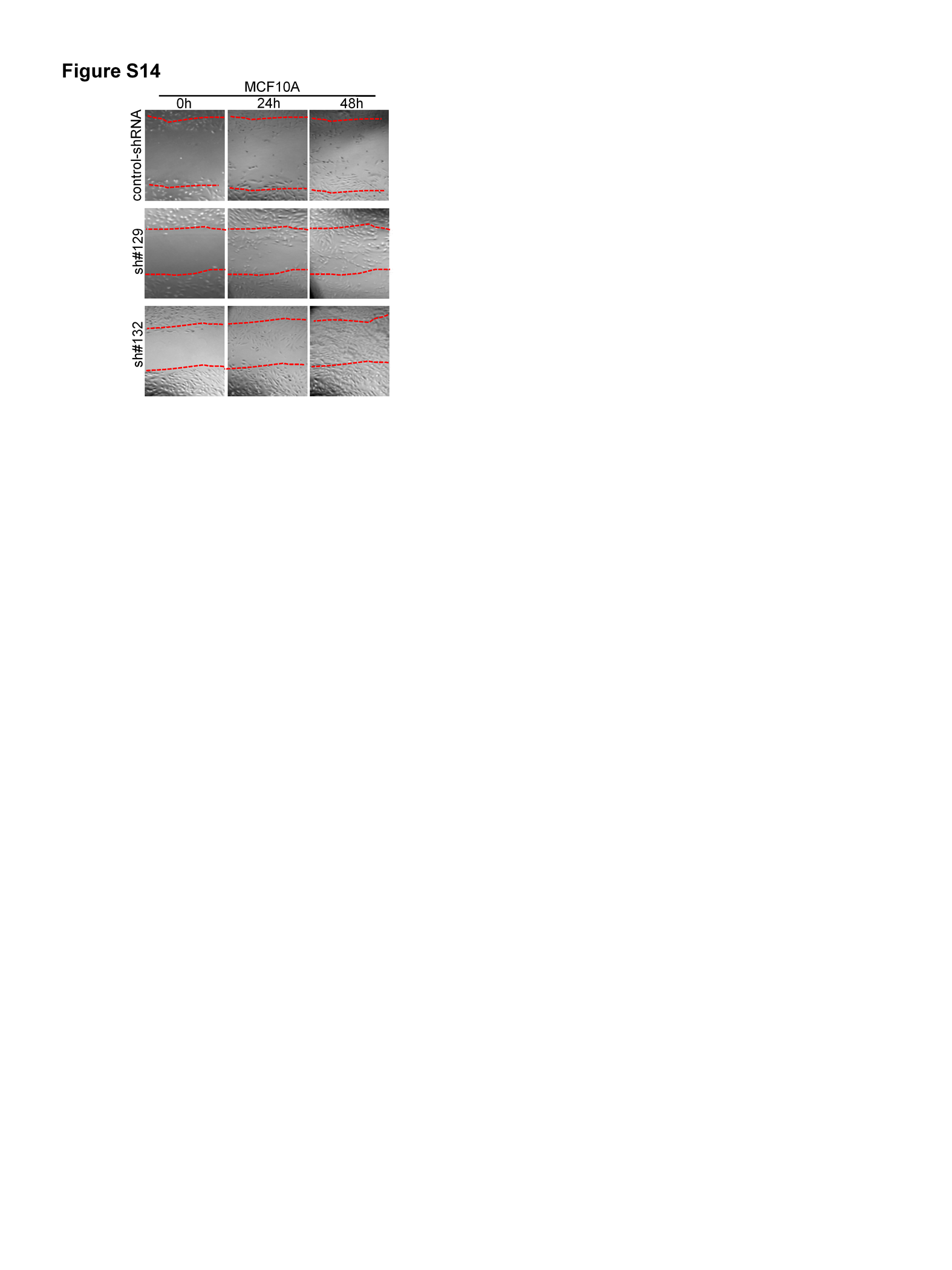

Supplement: Figure S14 — Wound-healing assay using control MCF10A cells or cells transduced with different shRNA against 14-3-3σ. (TIF) [file pone.0038347.s014.tif]
